# Supplementary material for: A Meiotic Checkpoint Alters Repair Partner Bias to Permit Inter-sister Repair of Persistent DSBs
Source: Cell Rep. 2019 Jan 15;26(3):775–787.e5. doi: 10.1016/j.celrep.2018.12.074 (PMC6334227; doi:10.1016/j.celrep.2018.12.074)
Supplement: Document S1. Figures S1–S7 and Table S1 [file mmc1.pdf]

Cell Reports, Volume *26*

Supplemental Information

A Meiotic Checkpoint Alters Repair Partner Bias

to Permit Inter-sister Repair of Persistent DSBs

Tatiana Garcia-Muse, U. Galindo-Diaz, M. Garcia-Rubio, J.S. Martin, J. Polanowska, N. O'Reilly, A. Aguilera, and Simon J. Boulton

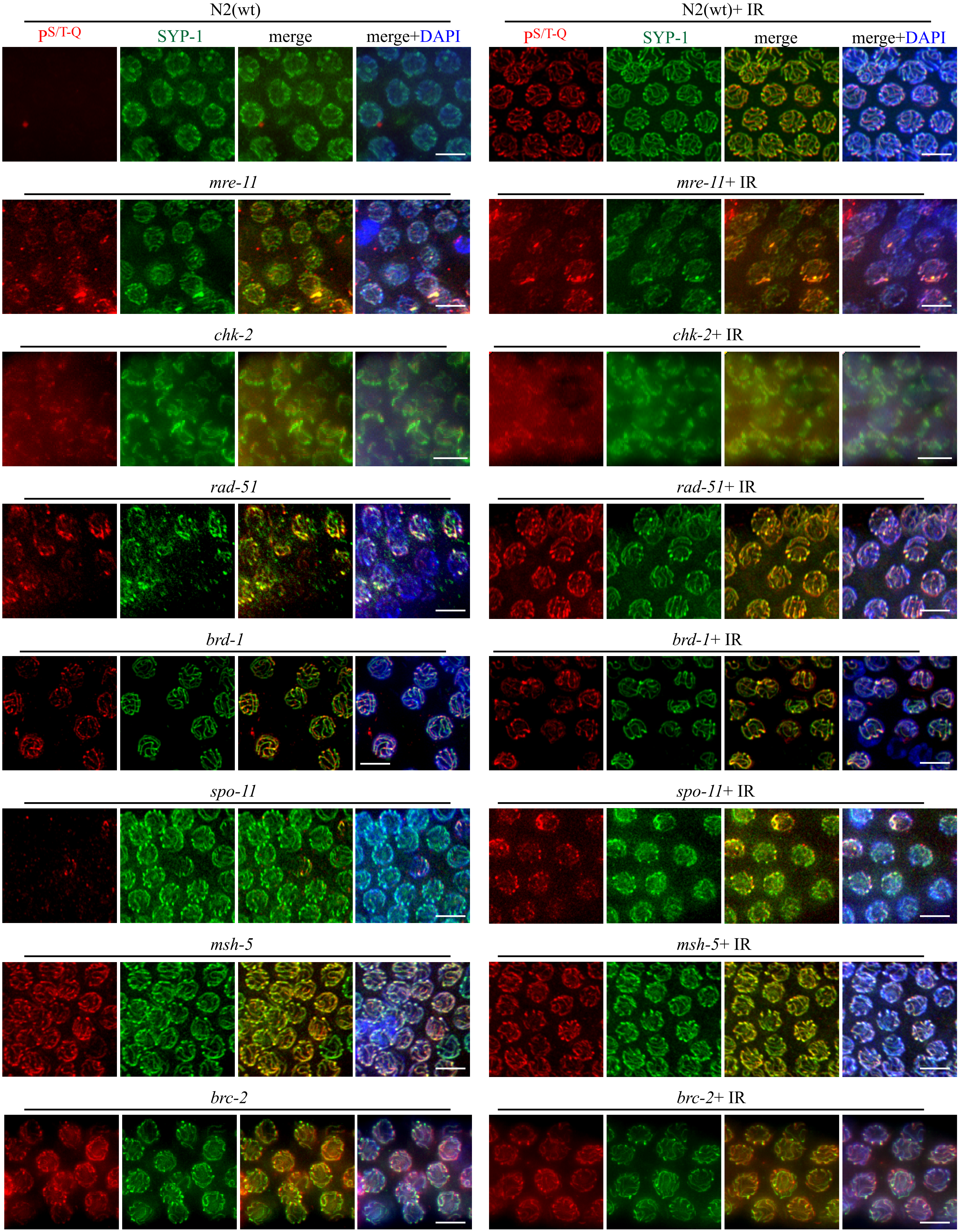

Figure S1. $P^{S/T-Q}$ phosphorylation in DNA repair and DNA damage response mutants, Related to Figure 1. Representative images of the pachytene region from the indicated strains fixed germlines immunostained with anti-$P^{S/T-Q}$ and Synaptonemal Complex protein SYP-1 antibodies and counterstained with DAPI without DNA damage or 1 hour post 75Gy. Scale bar 5μm.

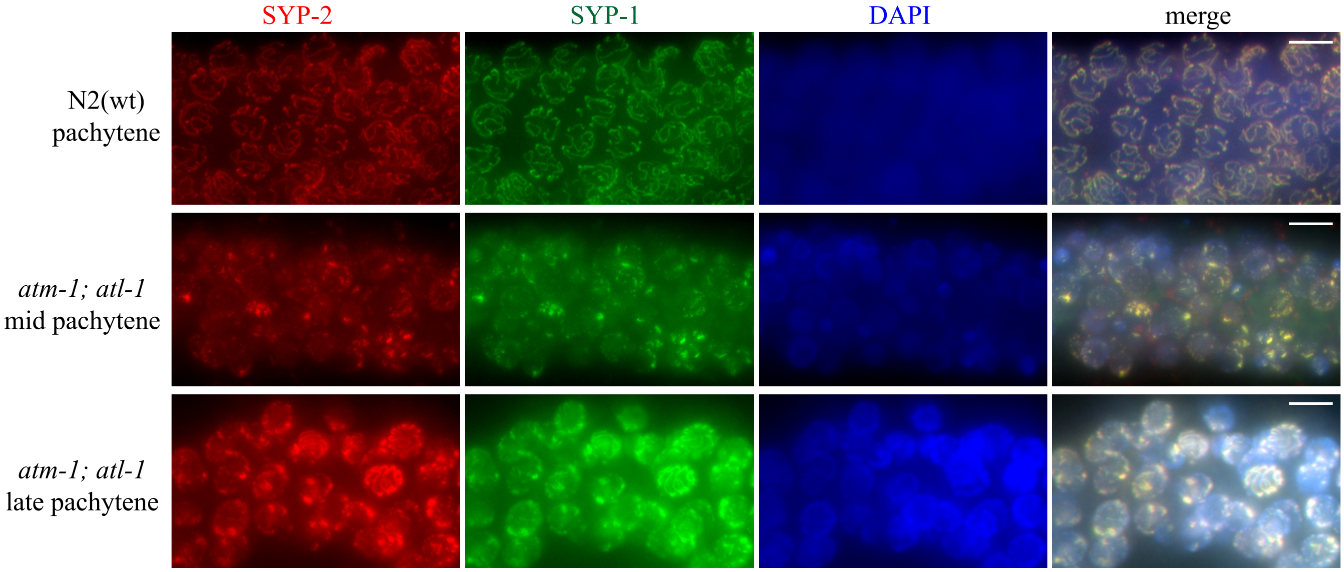

Figure S2. Synaptonelmal Complex is not well assembled in the *atm-1; atl-1* mutant, Related to Figure 1. Representative images of the pachytene region from the indicated strains fixed germlines immunostained with Synaptonemal Complex proteins SYP-1 and SYP-2 antibodies counterstained with DAPI. Scale bar 5µm.

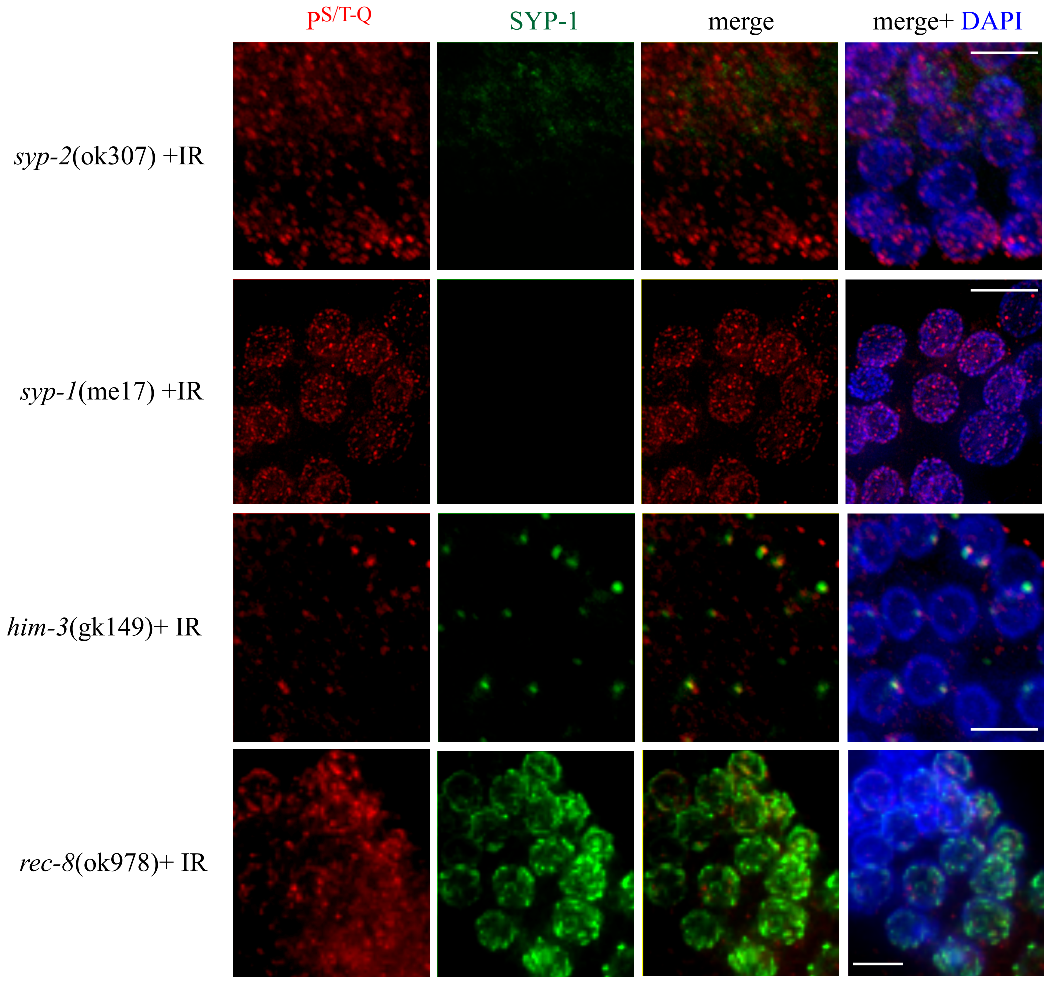

Figure S3. Single mutant screening for the target of the ATM/ATR phosphorylation in response to DNA damage, Related to Figure 2. Representative images of the pachytene region from the indicated strains fixed germlines immunostained with anti-P$^{S/T-Q}$ and Synaptonemal Complex protein SYP-1 antibodies counterstained with DAPI 1 hour post 75Gy. Scale bar 5µm.

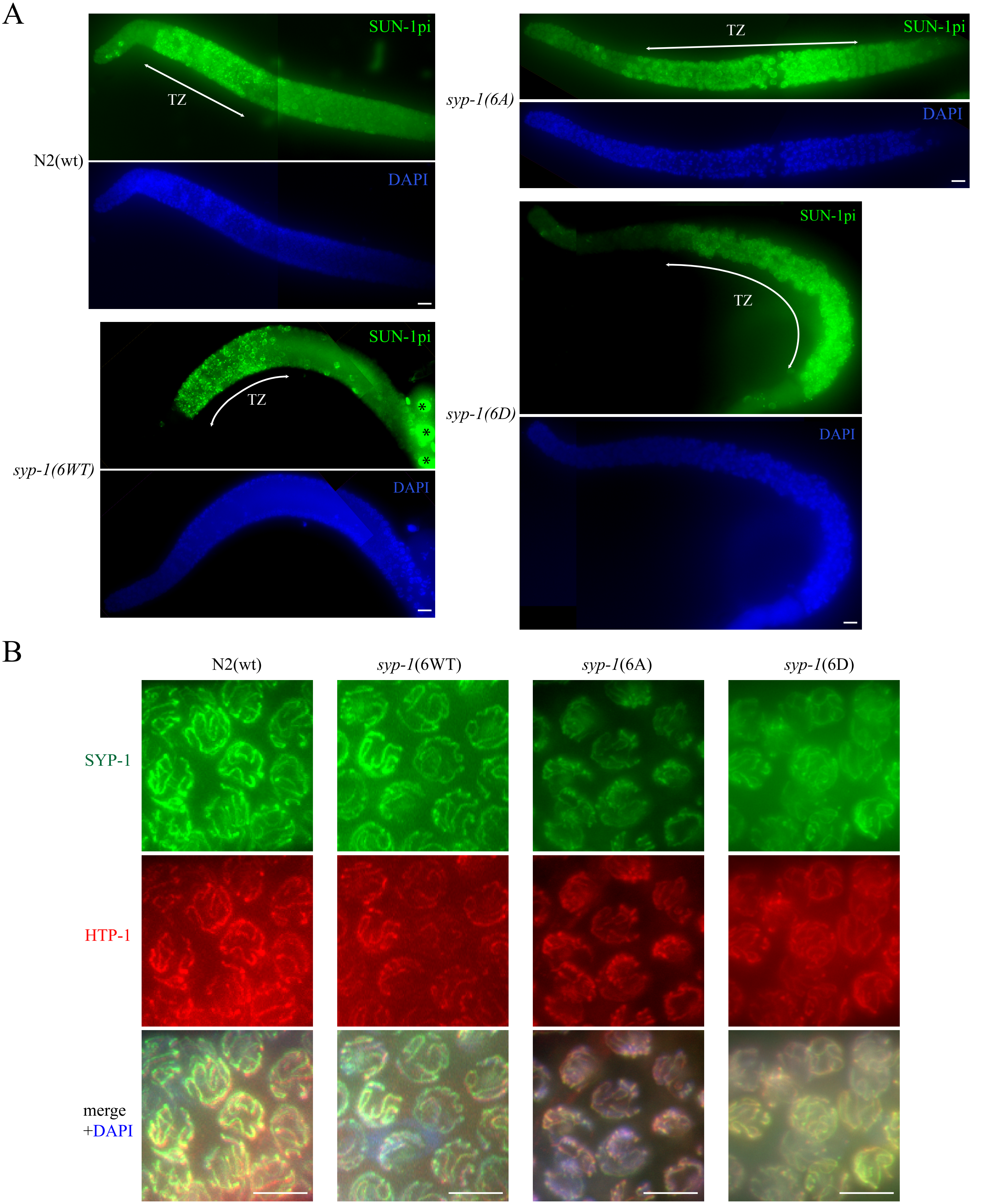

Figure S4. Synaptonemal Complex (SC) assembly in the *syp-1* phosphorylation alleles, Related to Figure 3. (A) Representative images of fixed germlines from the indicated strains immunostained with SUN-1pi antibody, marker of transition zone, and counterstained with DAPI. Scale bar 10µm. Images of the complete germilines were stitched together manually from two pictures that overlap. * Strong signal at diakinesis nuclei **(B)** Representative images of the pachytene region from the indicated strains fixed germlines immunostained with SYP-1 and HTP-1 antibodies (central and lateral elements of the SC, respectively) and counterstained with DAPI. Scale bar 5µm.

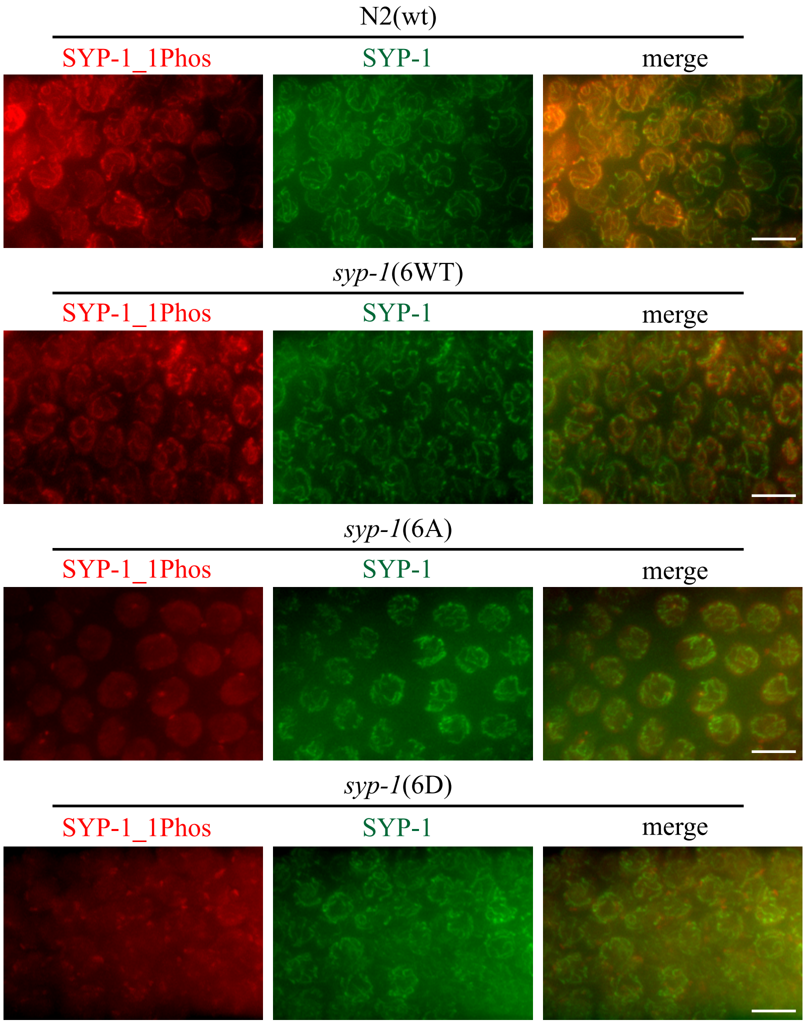

Figure S5. Phospho-antibodies in the *syp-1* phosphorylation alleles, Related to Figure 4. Representative images of the pachytene region from the indicated strains fixed germlines immunostained with SYP-1_1Phos and Synaptonemal Complex protein SYP-1 antibodies and counterstained with DAPI. Scale bar 5µm.

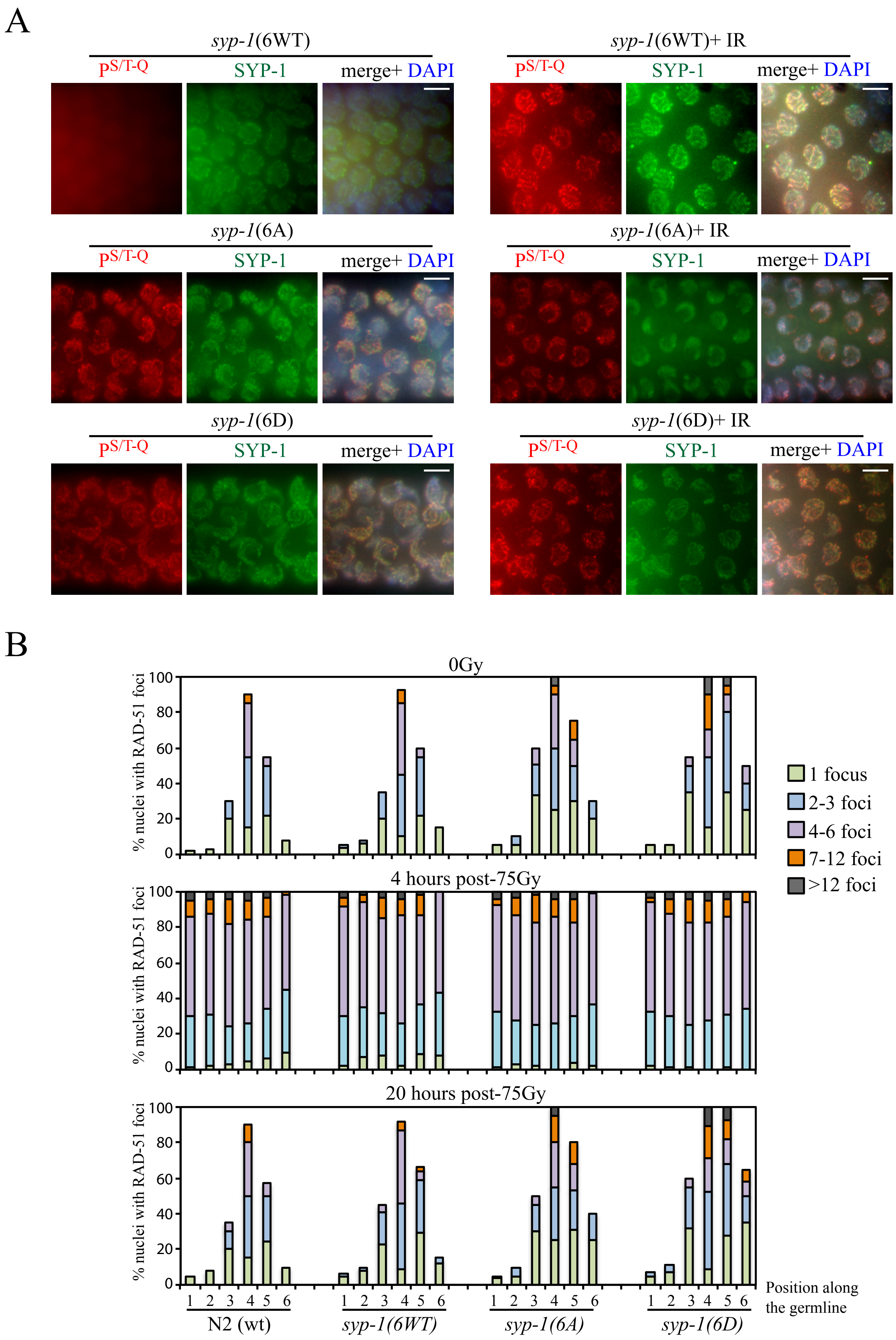

Figure S6. DNA damage response in the *syp-1* phosphorylation alleles, Related to Figure 5. (A) Representative images of the pachytene region from the indicated strains fixed germlines immunostained with anti-$P^{S/T-Q}$ and Synaptonemal Complex protein SYP-1 antibodies counterstained with DAPI without damage (left) or after 1 hour 75Gy (right). Scale bar 5µm. **(B)** Quantitation of RAD-51 focus formation in the indicated strains in normal conditions (top), or 4 (middle) and 20 (bottom) hours post 75Gy. At least fifteen gonads were analysed in each condition and ten nuclei were scored in each zone (mitotic region (1), transition zone (2), early-mid-late pachytene regions (3-4-5) and diplotene-diakinesis regions (6)).

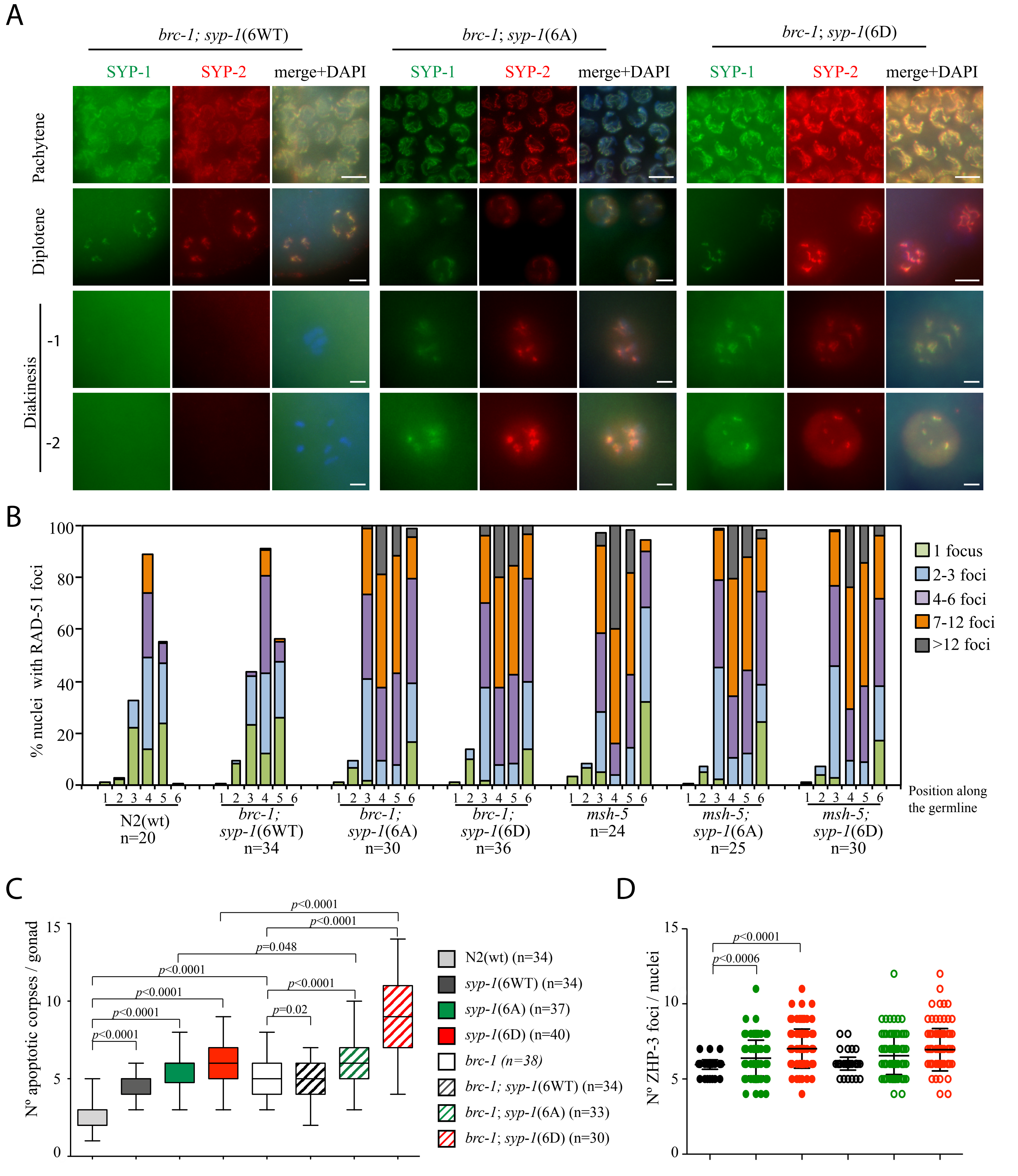

Figure S7. ***brc-1; syp-1* phosphorylation alleles, Related to Figure 6. (A)** Representative images of the indicated regions from the indicated strains fixed germlines immunostained with Synaptonemal Complex proteins SYP-1 and SYP-2 antibodies counterstained with DAPI. Scale bar 5µm. **(B)** Quantitation of RAD-51 focus formation in the indicated strains. (n) indicates the number of gonads analysed and ten nuclei were scored in each zone (mitotic region (1), transition zone (2), early-mid-late pachytene regions (3-4-5) and diplotene-diakinesis regions (6)). **(C)** Quantation of apoptotic corpses of the indicated strains. (n) indicates the number of animals analysed. Graph shows box and whiskers from minimun to maximun. P value for Paired T-test stadistical analysis is shown. **(D)** Quantation of ZHP-3 foci the indicated strains (120 late pachytene/diplotene nuclei were counted for each strain). Graph shows mean with SD. P value for Paired T-test stadistical analysis is shown.

Table S1. List of primers used in this study, Related to STAR Methods.

Primer	Sequence (5'-3')	Use
SYP1mos1.GWF	GGGGACAAGTTTGTACAAAAAAGCAGGCTGGGAATTTTGC GTATTTTCAAATTT	Cloning
SYP1mos1ter.GWR	GGGGACCACTTTGTACAAGAAAGCTGGGTAAACAGCGATT TTCAAATGTCCTGAT	Cloning
SYP-1site3&4-A-F	GTATTCTCGGCGCCATTGATGGCGGCCGCACCACTTGCCGC AGCTGCCAGACCGTTGAAACGAGCTCGTGCGGCCGATCG	Cloning
SYP-1site3&4-A-R	CGATCGGCCGCACGAGCTCGTTTCAACGGTCTGGCAGCTGC GGCAAGTGGTGCGGCCGCCATCAATGGCGCCGAGAATAC	Cloning
SYP-1site3&4-D-F	TTCTCGGCGCCATTGATGGATGACGATCCACTTGACGCAGC TGACAGACCGTTGAAACGAGATCGTGCGGCCGAT	Cloning
SYP-1site3&4-D-R	ATCGGCCGCACGATCTCGTTTCAACGGTCTGTCAGCTGCGT CAAGTGGATCGTCATCCATCAATGGCGCCGAGAA	Cloning
SYP-1 SQ1	CGGTGTTTGCGGCCTCCGCTC	Sequencing & genotyping
SYP-1 SQ3	GAATTGCAACTTGTCTCTGTC	Sequencing
SYP-1 SQ5	AGCAAATGAGGGAAGATTGCG	Sequencing & genotyping
SYP-1 SQ7	ACTATGATAAGCAAATCAAGG	Sequencing & genotyping
Ti5605E1	ATTCTTGAAGACGACGAGCC	Genotyping
Ti5605E2	TTTTCTCAGTTGTGATACGG	Genotyping
Ti5605E3	CACATCTTTTCTGGCTCTGC	Genotyping
Ti5605E4	TTCAGCCAAGTTACAGGCCG	Genotyping
Ti5605I1	TCAGAGCATTGCTTATCTCG	Genotyping
Ti5605I2	TGTAGTTATACAGAAGACCG	Genotyping
Ti5605I3	TCTGTAAAATATTATCATGG	Genotyping

Ti5605I4	GATCAGCCTCGTTGTCGACC	Genotyping
Cb unc-119 F	GCCGAGCAACAACAATCGATTCCACCCGGC	Genotyping
Cb unc-119 R	GCTTTGTTGTGCATGACGAGTTTGTTATCG	Genotyping
SYP-1 SQ-E1	AGGCAATCTGCAAATTTGCG	Genotyping
SYP-1 SQ-E2	CTGAGAACGGAGTGTGACGC	Genotyping
SYP-1 SQ2	AGTTTTCCCTCTTCGAGCGC	Genotyping
BRC-1 E1	CTCCGTAGCTTGAAGTCTCA	Genotyping
BRC-1 E2	TTTCGGTGGCGCCACATGGA	Genotyping
BRC-1 I1	AATATAGGCACCGGCGGGGA	Genotyping
BRC-1 I2	TGTCGCATCGTCGGCATTAA	Genotyping
BRC-1 E3	CCAGTGGTATGCTTTTTGGC	Genotyping
BRC-1 I3	CTGACTGAAAATCATAGCGG	Genotyping
MSH-5 I1	AGCTTGACCGCCGTCGCCTTACCCCG	Genotyping
MSH-5 I2	TCATCGAACTGTCATGGGACCCGGCC	Genotyping
MSH-5 I3	CGATATTTCAGAAGATCTTG	Genotyping
MSH-5 I4	CCTCTGATGTTCCTTCTTCTG	Genotyping
