# Supplementary material for: A Meiotic Checkpoint Alters Repair Partner Bias to Permit Inter-sister Repair of Persistent DSBs
Source: Cell Rep. 2019 Jan 15;26(3):775–787.e5. doi: 10.1016/j.celrep.2018.12.074 (PMC6334227; doi:10.1016/j.celrep.2018.12.074)
Supplement: Document S2. Article plus Supplemental Information [file mmc2.pdf]

Cell Reports

Article

A Meiotic Checkpoint Alters Repair Partner Bias to Permit Inter-sister Repair of Persistent DSBs

Graphical Abstract

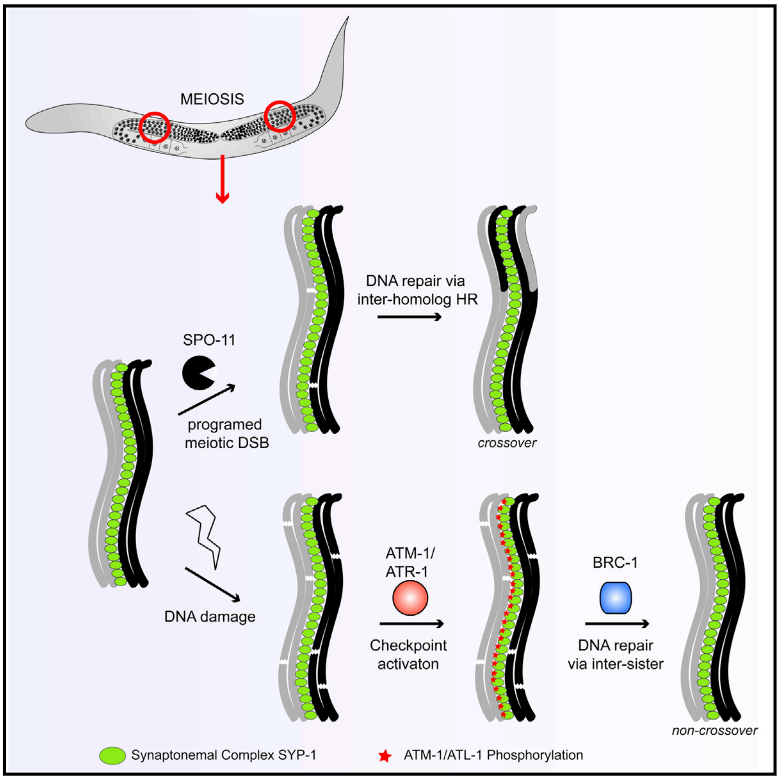

Highlights

- Meiotic DNA damage triggers phosphorylation of the synaptonemal complex (SC)
- ATM-ATR kinases phosphorylate the SC in response to excessive meiotic DSBs
- SC phosphorylation channels DNA repair to the sister chromatid

Authors

Tatiana Garcia-Muse, U. Galindo-Diaz, M. Garcia-Rubio, ..., N. O'Reilly, A. Aguilera, Simon J. Boulton

Correspondence

tatiana.muse@cabimer.es (T.G.-M.),
aguilo@us.es (A.A.),
simon.boulton@crick.ac.uk (S.J.B.)

In Brief

Garcia-Muse et al. show that the checkpoint kinases ATM and ATR respond to excessive or unrepaired meiotic DSBs by phosphorylating the core synaptonemal complex, which channels repair via the sister chromatid. These findings reveal a mechanism that switches repair partner bias to protect meiotic cells from unscheduled DNA breaks.

Garcia-Muse et al., 2019, Cell Reports *26*, 775–787
January 15, 2019 © 2018 The Author(s).
https://doi.org/10.1016/j.celrep.2018.12.074

CellPress

OPEN ACCESS
CellPress

A Meiotic Checkpoint Alters Repair Partner Bias to Permit Inter-sister Repair of Persistent DSBs

Tatiana Garcia-Muse,[1,2,4,*] U. Galindo-Diaz,[1] M. Garcia-Rubio,[1] J.S. Martin,[2] J. Polanowska,[2] N. O'Reilly,[3] A. Aguilera,[1,*] and Simon J. Boulton[2,3,*]
[1]Centro Andaluz de Biología Molecular y Medicina Regenerativa-CABIMER, Universidad de Sevilla-CSIC-Universidad Pablo de Olavide, Av. Américo Vespucio 24, 41092 Seville, Spain
[2]Clare Hall Laboratories, Blanche Lane, South Mimms EN6 3LD, UK
[3]DSB Repair Metabolism Laboratory, The Francis Crick Institute, Midland Road, London, UK
[4]Lead Contact
*Correspondence: tatiana.muse@cabimer.es (T.G.-M.), aguilo@us.es (A.A.), simon.boulton@crick.ac.uk (S.J.B.)
https://doi.org/10.1016/j.celrep.2018.12.074

SUMMARY

Accurate meiotic chromosome segregation critically depends on the formation of inter-homolog crossovers initiated by double-strand breaks (DSBs). Inaccuracies in this process can drive aneuploidy and developmental defects, but how meiotic cells are protected from unscheduled DNA breaks remains unexplored. Here we define a checkpoint response to persistent meiotic DSBs in *C. elegans* that phosphorylates the synaptonemal complex (SC) to switch repair partner from the homolog to the sister chromatid. A key target of this response is the core SC component SYP-1, which is phosphorylated in response to ionizing radiation (IR) or unrepaired meiotic DSBs. Failure to phosphorylate (*syp-1^{6A}*) or dephosphorylate (*syp-1^{6D}*) SYP-1 in response to DNA damage results in chromosome non-dysjunction, hyper-sensitivity to IR-induced DSBs, and synthetic lethality with loss of *brc-1^{BRCA1}*. Since BRC-1 is required for inter-sister repair, these observations reveal that checkpoint-dependent SYP-1 phosphorylation safeguards the germline against persistent meiotic DSBs by channelling repair to the sister chromatid.

INTRODUCTION

The formation of interhomolog crossovers by meiotic recombination is essential for the faithful segregation of homologous chromosomes necessary for the production of gametes for sexual reproduction. Crossovers are initiated by programmed DNA double-strand breaks (DSBs), whose repair, within the context of the synaptonemal complex (SC), visibly manifest as chiasmata at diakinesis. Inaccuracy in this process can produce aneuploidy, which results in embryonic lethality or pronounced developmental defects (Siegel and Amon, 2012).

Caenorhabditis elegans is a powerful model to study meiosis, as its germline is spatially organized with respect to the different phases of meiotic prophase I. The apical tip of the germline contains mitotic nuclei that undergo DNA replication prior to entry into meiosis. Adjacent to the mitotic compartment is the transition zone where homologous chromosomes align and pair, which precedes programmed meiotic DSB formation and inter-homolog recombination. By early pachytene, synapsis is complete with the SC assembled along the entire length of paired homologous chromosomes (Hillers et al., 2017). In contrast to most species, *C. elegans* homologous chromosome pairing is directed by pairing centers (PCs) (Villeneuve, 1994) that constitute binding sites for chromosome-specific HIM-ZIM zinc-finger proteins, which facilitate pairing through interactions with components of the nuclear periphery (Harper et al., 2011; Labella et al., 2011; Phillips and Dernburg, 2006; Phillips et al., 2005). Once correct pairing is achieved, homologous chromosome synapsis occurs via SC assembly.

The SC is a highly conserved proteinaceous structure that consists of a central region connecting two lateral or axial elements, which interact with the homologs. In *C. elegans*, there are four components that constitute the central SC region, SYP-1, SYP-2, SYP-3, and SYP-4, which are completely interdependent for SC assembly (Colaiácovo et al., 2003; MacQueen et al., 2002; Smolikov et al., 2007, 2009). Current data suggest that SYP-1, SYP-2, and SYP-3 are located in the middle of the central region, while SYP-3 links to SYP-1, SYP-4, and components of the lateral elements (Schild-Prüfert et al., 2011). Several factors affect SC assembly, including CHK-2 kinase, which is required for initial pairing between homologous chromosomes as well as for crossover formation (Alpi et al., 2003; MacQueen and Villeneuve, 2001). In contrast to wild-type worms, which present 6 bivalents at diakinesis, mutants defective for SC formation manifest 12 univalents due to the lack of crossover formation and the resulting chiasmata. While the SC primary role is to stabilize pairing interactions between homologs, it has also been shown to promote normal levels of crossover (Hayashi et al., 2010; Libuda et al., 2013).

Programmed meiotic DSBs are generated by the conserved Spo11 endonuclease across the genome (Keeney et al., 1997). These DSBs are repaired by homologous recombination (HR), and they require many of the enzymatic activities needed for HR-mediated repair of mitotic DNA damage. These include the MRE-11 nuclease for DSB resection and the RAD-51 recombinase for strand invasion into homologous duplex DNA (Heyer et al., 2010; Lui and Colaiácovo, 2013). The use of the

This is an open access article under the CC BY license (http://creativecommons.org/licenses/by/4.0/).

homologous chromosome as a template for DSB repair is regulated during pachytene, mainly by lateral SC components and through the inhibition of sister chromatid repair (Couteau et al., 2004; Martinez-Perez and Villeneuve, 2005). Meiotic DSBs induced in *syp-1* and *syp-2* mutants cannot be repaired through the homologous chromosome, and, hence, they persist until the barrier to sister chromatid repair is removed later in prophase (Colaiácovo et al., 2003). While dispensable for inter-homolog repair, BRC-1, the worm homolog of breast cancer tumor suppressor gene BRCA1, is essential for inter-sister DSB repair (Adamo et al., 2008; Boulton et al., 2004). Indeed, in a *syp-2* mutant background in which inter-homolog crossover formation is abolished, inactivation of sister chromatid repair by *brc-1* mutation leads to chromosome fragmentation at diakinesis (Adamo et al., 2008).

The DNA damage-responsive kinases ATM (ataxia-telangiectasia-mutated) and ATR (ataxia-telangiectasia-related) play central roles in DSB sensing and repair in mitotic cells (Abraham, 2001; Kastan and Bartek, 2004; Shiloh, 2001). ATM and ATR kinases also localize to meiotic chromosomes and have been implicated in promoting HR, repair template choice, and crossover control (MacQueen and Hochwagen, 2011). In mice, the loss of ATM leads to infertility due to meiotic defects, including meiotic DSB repair impairment since it can be rescued by crossing with heterozygous spo11 mice, which have reduced DSB formation (Keeney et al., 2014; Lange et al., 2011). ATR localizes to sex chromosomes, where it is involved in X chromosome inactivation and sex body formation, and it also localizes to unsynapsed chromosomes, where it plays a role activating the synapsis and homolog pairing checkpoints (MacQueen and Hochwagen, 2011). Budding yeast ATR, Mec1, is essential for meiosis, and it functions in promoting inter-homolog repair and regulating the number and distribution of cross-overs (COs). Both Mec1^{ATR} and Tel1^{ATM} promote inter-homolog recombination in meiosis via Hop1 phosphorylation (Carballo et al., 2008), and they suppress clustering of SPO11-dependent DSBs to ensure that crossover recombination is optimally dispersed along meiotic chromosomes (Gray et al., 2013). In *C. elegans*, the ATR kinase ATL-1 is essential for mitotic cell-cycle arrest and the induction of apoptosis in response to DNA damage, but it shows no obvious meiotic defects in SC assembly or crossover formation (Garcia-Muse and Boulton, 2005). ATM-1, on the other hand, plays a role in promoting localized desynapsis in response to DNA damage (Couteau and Zetka, 2011).

Here we investigated how the meiotic germline of *C. elegans* responds to and is protected from exogenous or persistent DNA damage. We present evidence that *C. elegans* ATM and ATR function redundantly as part of a meiotic checkpoint that responds to ionizing radiation (IR)-induced DSBs or persistent meiotic DSBs by phosphorylating core SC components to alter DSB repair partner bias. Using peptide array technology, we identified a cluster of DNA damage-induced phosphorylation sites in the core SC protein SYP-1, and we generated the corresponding non-phosphorylatable (SYP-1^{6A}) and phosphomimetic (SYP-1^{6D}) mutants to determine the importance of this modification *in vivo*. While both mutants complement the embryonic lethality of the *syp-1*(*me17*) null allele and exhibit normal pairing and synapsis, failure to regulate the phosphorylation state of SYP-1 confers sensitivity to exogenous DNA damage and synthetic lethality with *brc-1* mutants. Since BRC-1 is essential for inter-sister repair, our results support a critical role for damage-induced SYP-1 phosphorylation in promoting a switch in repair partner bias to allow repair of excessive or persistent meiotic DSBs via the sister chromatid. Hence, our work reveals a meiotic checkpoint that acts to protect the germline from unscheduled DNA damage and genetic instability.

RESULTS

Meiotic ATM-ATR Phosphorylation in Response to DNA Damage

To directly visualize phosphorylation events induced by ATM-ATR kinases within the germline, we performed immunostaining with a phospho-(Ser/Thr) ATM-ATR substrate motif antibody (P$^{S/T-Q}$) (Abraham, 2001). This exploited a unique feature of the *C. elegans* germline, which is spatially polarized in a distal-to-proximal manner with respect to proliferation and progression through meiotic prophase. Germline staining for P$^{S/T-Q}$ in N2 wild-type animals was largely absent under normal growth condition, although a low signal was observed occasionally in late pachytene, when nuclei can undergo apoptosis. In contrast, animals subjected to hydroxyurea (HU) or IR displayed robust P$^{S/T-Q}$ staining in the mitotic nuclei of the pre-meiotic zone of the germline, consistent with the established response of ATM-ATR in mitotic cells (data not shown). Distal to the P$^{S/T-Q}$ staining in the mitotic zone, IR treatment also induced an unexpected P$^{S/T-Q}$ signal in zygotene and pachytene nuclei, which localized between and along the length of paired chromosomes (Figure 1A). This P$^{S/T-Q}$ staining resembled the SC and axial element that hold homologous chromosomes together during meiotic prophase. Importantly, the SC and axial element pattern of P$^{S/T-Q}$ staining was abolished by phosphatase treatment, confirming that the staining corresponds to a phosphorylation event (Figure 1B).

To determine the genetic requirements for the IR-induced meiotic P$^{S/T-Q}$ signal, we first subjected worms to caffeine treatment, an inhibitor of the ATM and ATR family of kinases (Blasina et al., 1999; Hall-Jackson et al., 1999; Sarkaria et al., 1999). Animals subjected to growth in caffeine no longer displayed the IR-induced P$^{S/T-Q}$ signal in either mitotic or meiotic nuclei, suggestive of a role for ATM and/or ATR in this response (Figure 1B). Surprisingly, however, single *atm-1*(*gk186*) and *atl-1*(*tm853*) null mutants (Garcia-Muse and Boulton, 2005; Parusel et al., 2006) maintained the IR-induced P$^{S/T-Q}$ staining throughout the germline after IR treatment (Figure 1C). Given this result, we considered the possibility that ATM and ATR may act redundantly in this response. Indeed, the IR-induced P$^{S/T-Q}$ signal was greatly reduced in *atm-1*;*atl-1* double mutants, implying that this response can be elicited by either checkpoint kinase (Figure 1C). Notably, when both checkpoint kinases were suppressed, either by treating the nematodes with caffeine or by their mutation inactivation, the SC was significantly altered (Figure 1B; Figure S2).

We next assessed IR-induced P$^{S/T-Q}$ staining in mutants implicated in meiotic DSB sensing, generation, and/or repair (*chk-2*, *mre-11*, *spo-11*, *rad-51*, *msh-5*, *brc-2*, *brc-1*, and *brd-1*). *chk-2* mutants are dispensable for the DNA damage

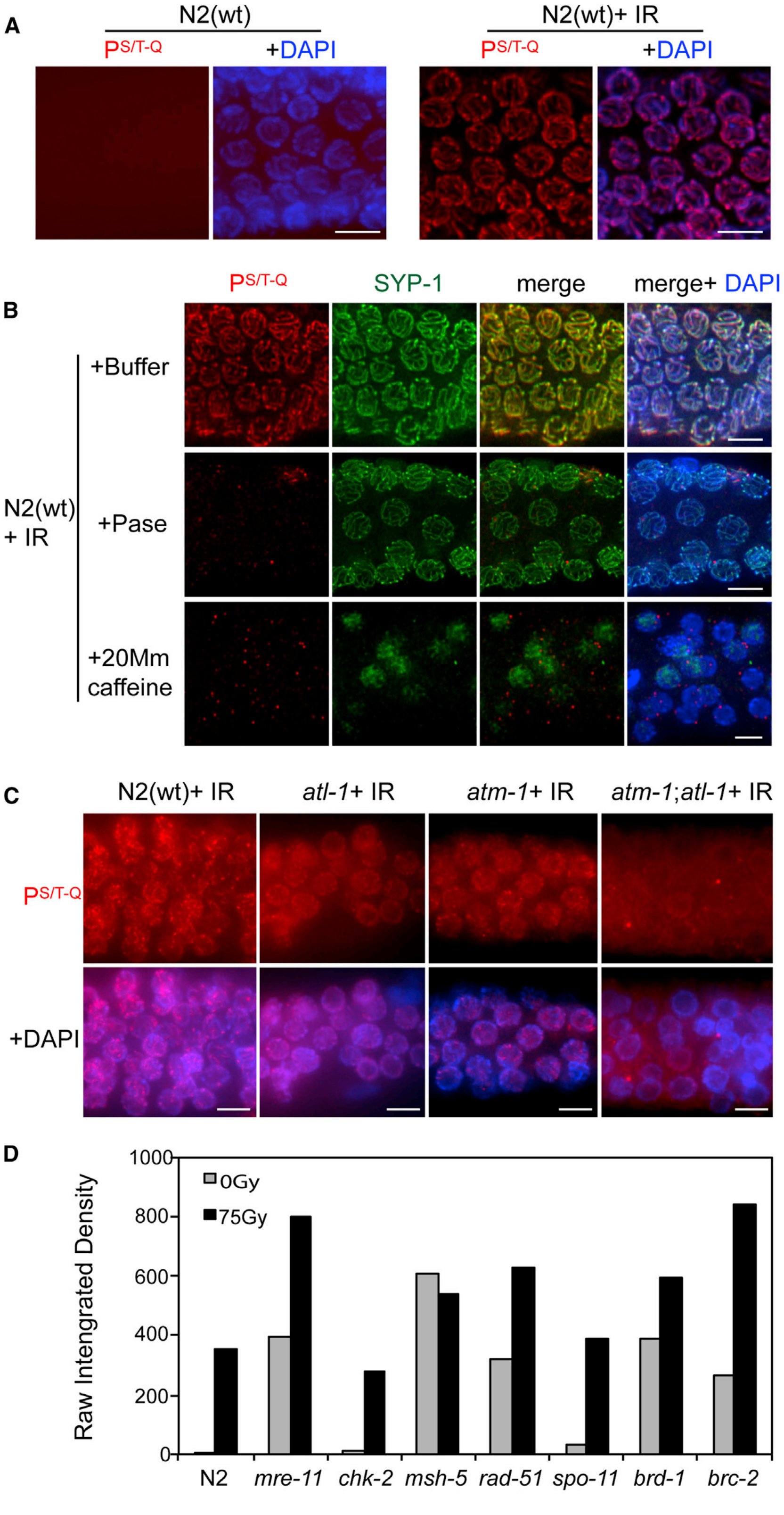

Figure 1. ATM-ATR-Dependent Phosphorylation in Response to DNA Damage

(A) Representative images of the meiotic region from N2(WT) fixed germlines immunostained with anti-$P^{S/T-Q}$ antibody and counterstained with DAPI without DNA damage (left) and 1 h after 75 Gy (right). Scale bar, 5 μm.

(B) Representative images of the meiotic region from N2(WT) fixed germlines immunostained with anti-$P^{S/T-Q}$ and SYP-1 antibodies and counterstained with DAPI 1 h after 75 Gy, previously incubated with buffer (top), phosphatase (middle), or with the animals previously grown in the presence of 20 mM caffeine for 4 h (bottom). Scale bar, 5 μm.

(C) Representative images of the meiotic region from the indicated strains' fixed germlines immunostained with anti-$P^{S/T-Q}$ and synaptonemal complex protein SYP-1 antibodies and counterstained with DAPI 1 h after 75 Gy. Scale bar, 5 μm.

(D) Quantification of $P^{S/T-Q}$ in the indicated strains in normal conditions (gray bars) or 20 h after 75 Gy (black bars). Graph shows intensity signal (arbitrary units, not normalized) determined by ImageJ software. 20–30 nuclei/germline from mid-pachytene were analyzed.

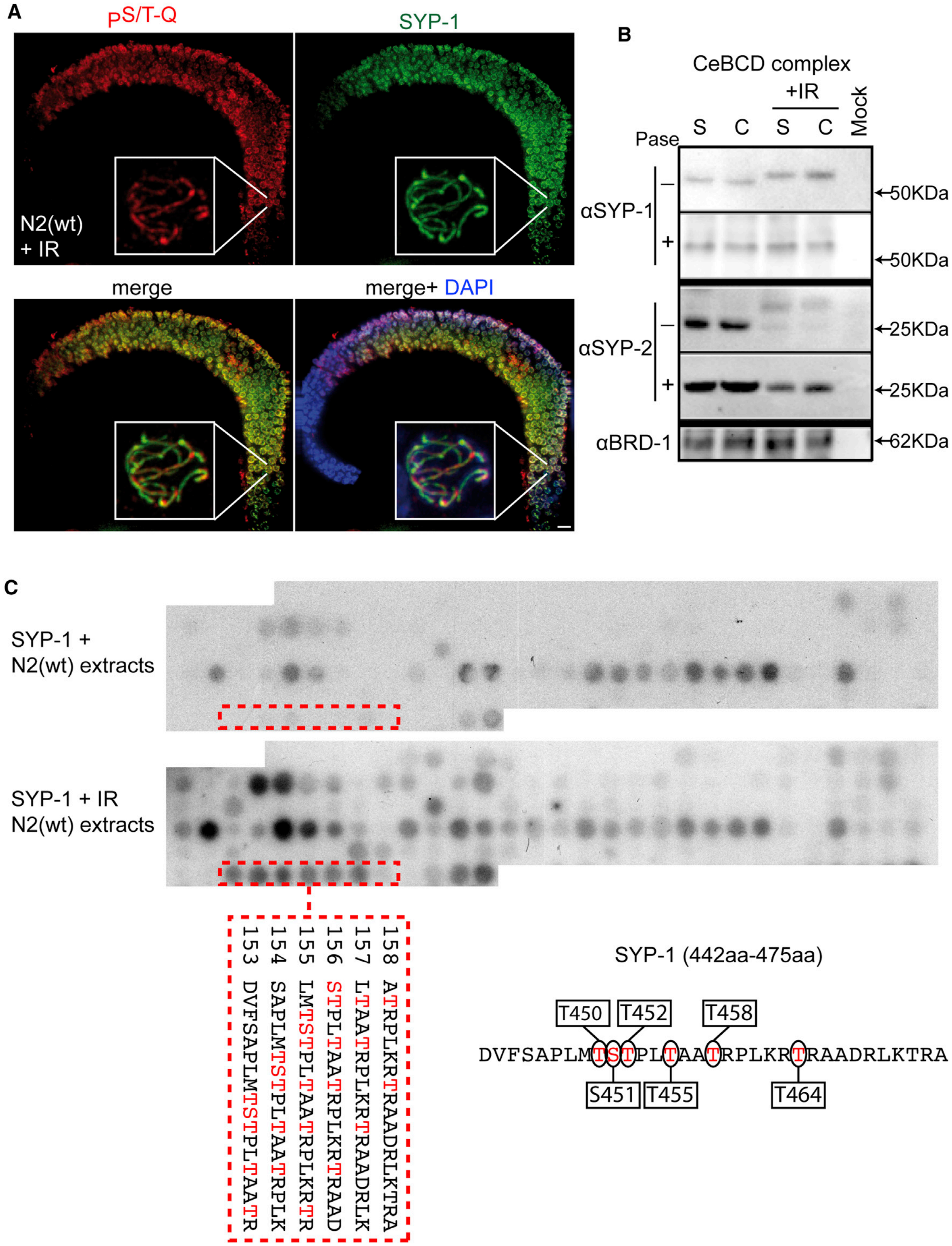

Figure 2. Meiotic Phosphorylation in Response to DNA Damage

(A) Representative images of whole N2(WT) fixed germlines immunostained with anti-P$^{S/T-Q}$ antibody and counterstained with DAPI 1 h after 75 Gy. Scale bar, 10 μm.

(B) Western blot using SYP-1, SYP-2, and BRC-1 antibodies of the mock purification and CeBCD complex following tandem immunoaffinity purification (S, soluble and C, chromatin bound, before and after IR treatment). Samples were treated or not with phosphatase.

(legend continued on next page)

response (DDR) checkpoint and displayed a normal response (Figures 1D and S1). IR-induced $P^{S/T-Q}$ staining was also detected in *mre-11*, *spo-11*, *rad-51*, *msh-5*, and *brd-1* mutant worms (Figures 1D and S1). Intriguingly, mutants defective for meiotic DSB repair, including *mre-11*, *rad-51*, *brc-2*, *msh-4*, and *brd-1*, exhibited robust $P^{S/T-Q}$ staining, resembling the SC and axial element in untreated conditions (i.e., without IR) (Figures 1D and S1). Since these mutants exhibited persistent meiotic DSBs, the data suggest that the meiotic checkpoint response is not limited to IR-induced DSBs but also extends to persistent meiotic DSBs that arise when repair is delayed or compromised. Hence, we propose that ATM-ATR respond to IR or persistent meiotic DSBs by inducing the phosphorylation of the meiotic target(s) that is situated in close proximity to the SC and axial element.

DNA Damage Phosphorylation Sites in SC Component SYP-1

Given the similarity of the meiotic $P^{S/T-Q}$ signal to the SC and axial element (MacQueen et al., 2002), we performed germline co-staining of IR-treated animals with $P^{S/T-Q}$ and SYP-1 (a central region component of the SC) antibodies, which revealed extensive co-localization along the majority of the SC (Figure 2A). Consistent with the target(s) for the meiotic checkpoint residing within the SC or axial element, IR-induced $P^{S/T-Q}$ staining was profoundly disrupted in the SC or axial element mutants, including *syp-1*(*me17*), *syp-2*(*ok307*), and *him-3*(*e1147*) or the cohesin mutant *rec-8*(*ok978*) (Colaiácovo et al., 2003; Hayashi et al., 2007; MacQueen et al., 2002; Pasierbek et al., 2001; Zetka et al., 1999) (Figure S3). Importantly, all tested mutants showed reduced, but not abolished, meiotic $P^{S/T-Q}$ signal after IR, suggesting that more than one protein is subject to phosphorylation as part of this response. Indeed, western blotting of N2 wild-type extracts before and after IR treatment for the core SC components SYP-1 and SYP-2, obtained after tandem immunoaffinity purification of CeBCD (*C. elegans* BRCA1/BARD) complex, revealed a mobility shift for both proteins after IR that was collapsed to the size of the untreated band with phosphatase (Figure 2B).

Our data raised the possibility that the core SC is a target for meiotic checkpoint-dependent phosphorylation in response to IR-induced and persistent meiotic DSBs. Hence, we sought to identify potential phosphorylation sites in SC proteins and particularly those induced by IR. To this end, we focused our efforts on SYP-1, and we designed peptide arrays comprising 18-mer peptides juxtaposed by 3 amino acids covering the entire length of the protein. The resulting SYP-1 peptide array was subjected to kinase assays using N2 wild-type extracts generated before and after 75-Gy IR treatment extracts and adenosine triphosphate (γ-^{32}ATP). In addition to putative constitutive phosphorylation sites present on the array irrespective of condition, we identified a cluster of serine and threonine residues between 450 and 464 amino acids of SYP-1 that were phosphorylated only in the extracts from IR-treated animals (Figure 2C).

To investigate the biological relevance of the damage-induced phosphorylation sites in the SYP-1 protein, we generated three transgenic lines using the mos1-mediated single copy insertion (MosSCI) system (Frøkjaer-Jensen et al., 2008), including: (1) a phospho mutant of *syp-1* in which the phosphorylated residues were changed to alanine (*syp-1*(*6A*)); (2) a phospho-mimetic *syp-1* mutant in which the phosphorylated residues were changed to aspartic acid (*syp-1*(*6D*)); and (3) a wild-type *syp-1* allele (*syp-1*(*6WT*)). The resulting transgenic lines were then crossed with the *syp-1*(*me17*) null mutant to eliminate endogenous SYP-1, leaving the transgenes as the only source of *syp-1* expression. *syp-1*(*me17*) mutants are defective for SC assembly, and, consequently, they exhibit an absence of chiasmata, increased chromosome non-dysjunction, and 95% embryotic lethality due to aneuploid gametes (MacQueen et al., 2002). We first tested if the wild-type *syp-1*(*6WT*) allele could complement the *syp-1*(*me17*) mutation by a survival assay in the *syp-1*(*6WT*) strain (Table 1), and indeed viability was rescued to 99.6% (n = 12), a value similar to N2(WT) worms. *syp-1*(*6A*) and *syp-1*(*6D*) strains also rescued the *syp-1* null phenotype but to a lesser extent, corresponding to 82.2% (n = 22) and 85.8% (n = 32) viability, respectively (Table 1).

Chromosome non-dysjunction in the *syp-1*(*me17*) null strain leads to a high incidence of males (38%) among the rare surviving progeny, because X chromosome ploidy determines sex in *C. elegans* (Him phenotype) (Hodgkin et al., 1979; MacQueen et al., 2002). The *syp-1*(*6WT*) allele complemented the *syp-1*(*me17*) null Him phenotype to wild-type levels of males (0.35%). Consistent with the partial rescue of viability, *syp-1*(*6A*) and *syp-1*(*6D*) strains exhibited 6.1% and 6.3% males, respectively (Table 1). Collectively, these data suggest that the failure to regulate the phosphorylated state of SYP-1 mildly alters normal meiosis, leading to reduced viability and an elevation in chromosome non-dysfunction.

Phosphorylation of SYP-1 Alters SC Disassembly

The *C. elegans* germline allows for temporal and spatial analyses of meiotic progression through prophase I (Hillers et al., 2017). Cytological analysis of fixed germlines isolated from *syp-1*(*6A*) and *syp-1*(*6D*) strains revealed that the transition zone is moderately extended when compared to *syp-1*(*6WT*) allele-complemented strains, as determined by nuclei morphology and immunofluorescence with SUN-1ph antibody (Penkner et al., 2009), a marker of the transition region (Figures 3A and S4A). Since extension of the transition zone can originate from defects in homologous chromosome pairing or synapsis, we examined homologous chromosome pairing by scoring one versus two HIM-8 foci, which specifically localizes to the pairing center on the X chromosome (Phillips et al., 2005). HIM-8 staining revealed

(C) *In vitro* phosphorylation of the SYP-1 peptide array by N2(WT) extracts without DNA damage (top) and with N2(WT) extracts after 75 Gy (bottom). Each of the 127 spots represents an 18-mer peptide fragment juxtaposed by three amino acids (aa) scanning the complete SYP-1 protein. Each peptide has a 15-amino acid overlap with the previous peptide and is numbered sequentially from the start codon. Positive serial spots (detected by autoradiography) corresponding to the specific DNA damage-phosphorylated region are boxed. The peptide sequences with specific DNA damage phosphorylation are shown with the possible phosphorylation residues highlighted in red. Scheme shows the phosphorylation site established by the peptide array data.

Table 1. Viability Analysis of syp-1 Mutant Alleles

Genotype	Average Brood ± SD (n)[a]	Percentage Viable Embryos (n)[b]	Percentage Larval Arrest (n)[c]	Percentage Male (n)[d]
N2(WT)	296.1 ± 8.8 (24)	99.9 (7,107)	0.03 (2)	0 (7,103)
syp-1(me17)	226.6 ± 60.1 (6)	4.4 (1,360)	25 (15)	31.6 (45)
syp-1(6WT); syp-1(me17)	264.2 ± 31.3 (12)	99.6 (3,170)	0.06 (2)	0.35 (3,158)
syp-1(6A); syp-1(me17)	253.8 ± 32.6 (22)	82.2 (5,584)	3.4 (143)	6.16 (4,450)
syp-1(6D); syp-1(me17)	201.4 ± 47.9 (32)	85.8 (6,446)	4.1 (227)	6.33 (5,306)
brc-1	282.1 ± 3.72 (7)	99.4 (1,975)	0.05 (1)	0.1 (1,961)
brc-1; syp-1(6WT); syp-1(me17)	209.4 ± 47.5 (19)	99.2 (3,978)	0.03 (1)	0 (3,944)
brc-1; syp-1(6A); syp-1(me17)	235.1 ± 48.4 (7)	32.9 (1,646)	4.05 (22)	6.7 (521)
brc-1; syp-1(6D); syp-1(me17)	201.6 ± 82.8.4 (28)	18.3 (5,810)	16.7 (178)	5.4 (885)

[a]Parentheses indicate the total number of singled hermaphrodites for which entire brood sizes were scored.
[b]Parentheses indicate the total number of fertilized eggs scored.
[c]Parentheses indicate the total number of <L4 worms.
[d]Parentheses indicate the total number of adults scored.

normal pairing between homologs in *syp-1*(*6A*) and *syp-1*(*6D*) strains in pachytene nuclei, comparable to *syp-1*(*6WT*) and N2(WT) strains (Figure 3B). We then examined homologous chromosome synapsis by immunostaining for the SC central region proteins SYP-1 and SYP-2 and for SC axial element HTP-1 (Colaiácovo et al., 2003; Martinez-Perez et al., 2008), and we observed that their localization between paired homologous chromosomes in the *syp-1*(*6WT*), *syp-1*(*6A*), and *syp-1*(*6D*) strains is indistinguishable from N2(WT), both in the transition zone and throughout pachytene (Figures 3A and S4A). Taken together, these results indicate that homologous pairing and synapsis upon entrance into meiosis are not affected in the *syp-1*(*6A*) and *syp-1*(*6D*) strains.

At the pachytene-diplotene transition, the SC central region components begin to disassemble from chromatin in early diakinesis, becoming progressively restricted to the mid-bivalent and then being completely removed from chromosomes by late diakinesis in −2 and −1 oocytes (Figure 3C). In contrast to N2(WT) and *syp-1*(*6WT*) strains, SC disassembly is modestly delayed in *syp-1*(*6A*) and *syp-1*(*6D*) mutants, as revealed by the persistent chromosome-associated SYP-1 and SYP-2 in −2 and −1 oocytes (Figure 4A). Since the *syp-1* mutant alleles exhibit a Him phenotype (high incidence of males) indicative of X chromosome non-disjunction (Table 1), we also analyzed chromosome morphology at diakinesis. In general, most of the diakinetic nuclei in the *syp-1*-complemented strains showed the normal N2(WT) complement of six bivalents, but with the occasional nuclei with 7 DAPI-stained bodies. This contrasts with the 12 univalents present at diakinesis in the *syp-1*(*me17*) mutant (MacQueen et al., 2002; Figures 4B and 4C). Collectively, these results indicate that chromosomes pair and undergo synapsis normally in the strains harboring the *syp-1*(*6A*) and *syp-1*(*6D*) alleles but disassembly of the SC is slightly delayed, which could explain the modest effect on X chromosome missegregation during meiosis.

While we were preparing this paper, phosphorylation of SYP-1 with a role in meiosis progression was described (Sato-Carlton et al., 2018). This prompted us to test if this modification is altered in our strains. Immunofluorescence with the T452_1-phos antibody showed the reported signal in N2(WT) and *syp-1*(*6WT*) germlines. In contrast, both mutant alleles abolished T452_1-phos staining, which was expected since the T452 is one of the residues substituted in our *syp-1*(*6A*) and *syp-1*(*6D*) alleles (Figure S5). Sato-Carlton and colleagues observed the same meiotic phenotypes, but their embryonic lethality data differ from ours. Importantly, in our *syp-1* alleles, we have mutated two additional residues, which likely explains the differences we see.

Delayed DNA Repair in *syp-1(6A)* and *syp-1(6D)* Alleles

Next, we assessed whether the phosphorylation status of SYP-1 impacts on the ability of worms to respond to exogenous DNA damage induced by IR. Following exposure to different doses of IR, we determined the IR sensitivity by scoring survival of the resulting F1 progeny 24–36 h after irradiation of L4 stage hermaphrodites (Craig et al., 2012). N2(WT) and *syp-1*(*6WT*) strains exhibited comparable survival rates of 80%, 55%, and 25% after irradiation with 50, 75, and 100 Gy, respectively (Figure 5A). Strikingly, the *syp-1*(*6D*) mutant strain showed heightened sensitivity corresponding to survival rates of 30%, 18%, and 8% after irradiation with 50, 75, and 100 Gy, respectively. The *syp-1*(*6A*) strain exhibited intermediate sensitivity between the N2(WT) and the *syp-1*(*6D*) mutant strains (Figure 5A). To confirm that the ATM-1/ATL-1 checkpoint response was still induced in our mutant alleles, we performed immunostaining with the $P^{S/T-Q}$ antibody. $P^{S/T-Q}$

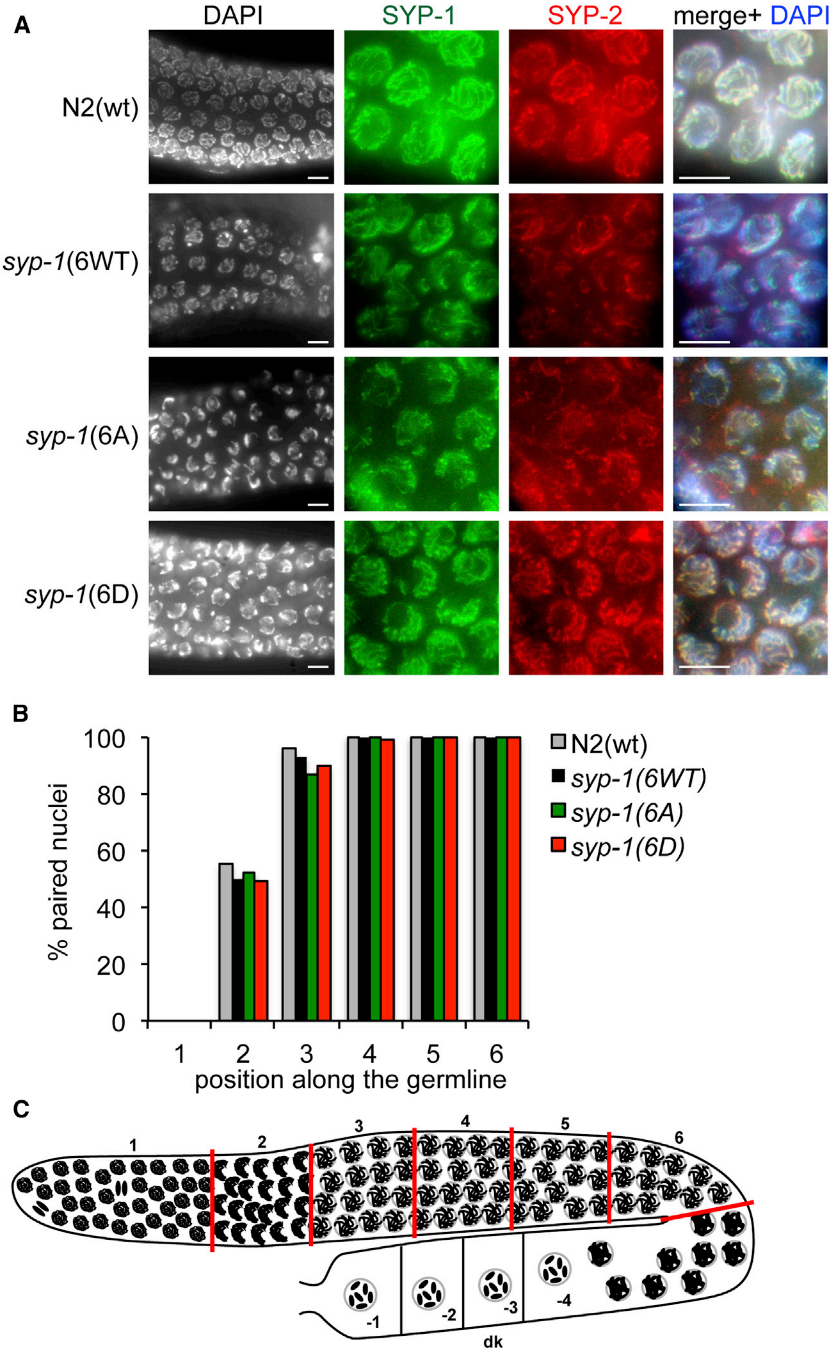

Figure 3. Synaptonemal Complex Assembly in *syp-1* Alleles

(A) Representative images of transition zone and pachytene region from the indicated strains' fixed germlines immunostained with synaptonemal complex protein SYP-1 and SYP-2 antibodies and counterstained with DAPI. Scale bar, 5 μm.

(B) Quantitation of pairing for chromosome X shown as the percentage of nuclei with paired signals in each zone shown in (C). Pairing of the X chromosome was visualized by immunofluorescence against HIM-8, which binds to the left end of the X chromosome at the *cis*-acting pairing center (PC). At least 15 gonads were scored for each genotype.

(C) Diagram of a hermaphrodite gonad, indicating the zones in which the pairing of HIM-8 signal (one foci versus two foci) was scored. 1, mitotic; 2, leptotene and zygotene; 3, early pachytene; 4 and 5, mid-pachytene; 6, late pachytene; 7, diplotene and diakineis.

staining was observed after IR treatment in the *syp-1*(*6WT*), *syp-1*(*6A*), and *syp-1*(*6D*) strains (Figure S6A). This was expected as SYP-1, SYP-2, and potentially many other meiotic substrates are phosphorylated in response to IR. Notably, both mutant alleles showed occasional $P^{S/T-Q}$ staining in the germline even without IR treatment, which might reflect a delay in processing DNA damage.

To monitor the repair of meiotic and irradiation-induced DSBs, we used an antibody against *C. elegans* RAD-51, which is essential for the strand invasion and exchange steps during HR (Alpi et al., 2003). During normal meiosis, RAD-51 foci are observed at sites of SPO-11-induced meiotic DSBs. In N2(WT) and *syp-1*(*6WT*) germlines, RAD-51 foci first appear in the transition zone and progressively increase in foci number in a given nucleus, reaching a maximum in mid-pachytene and finally disappearing in late pachytene. In the case of *syp-1*(*6A*) and *syp-1*(*6D*) germlines, we observed a modest increase in the number of nuclei with RAD-51 foci as well as the number of foci per nuclei (Figures 5B and S6B).

Next, we analyzed the number and distribution of RAD-51 foci after IR. In N2(WT) and *syp-1*(*6WT*) germlines at 20 h post-treatment with 75-Gy IR, we observed elevated levels of RAD-51 foci, which were resolved by late pachytene with comparable kinetics (Figures 5C and S6B). In contrast, a significant delay in DSB repair was observed in the *syp-1* mutant strains, which was particularly pronounced in the *syp-1*(*6D*) mutant, where RAD-51 foci persisted into diakinesis (Figures 5C and S6B). Since accumulation of unrepaired DNA damage leads to apoptosis, we also scored germ cell apoptosis in late L4 worms 12, 24, and 36 h after treatment with 75-Gy IR. Apoptotic corpses were significantly increased in *syp-1*(*6D*) mutant strains, increased when compared to N2(WT), *syp-1*(*6WT*), and *syp-1*(*6A*) (Figure 5D), which correlates with the delayed repair of DSBs observed in these strains. Taken together, these results indicate that the phosphorylation state of SYP-1 regulates the ability to process DNA damage in meiotic cells and is important for preventing genomic instability and apoptosis.

A

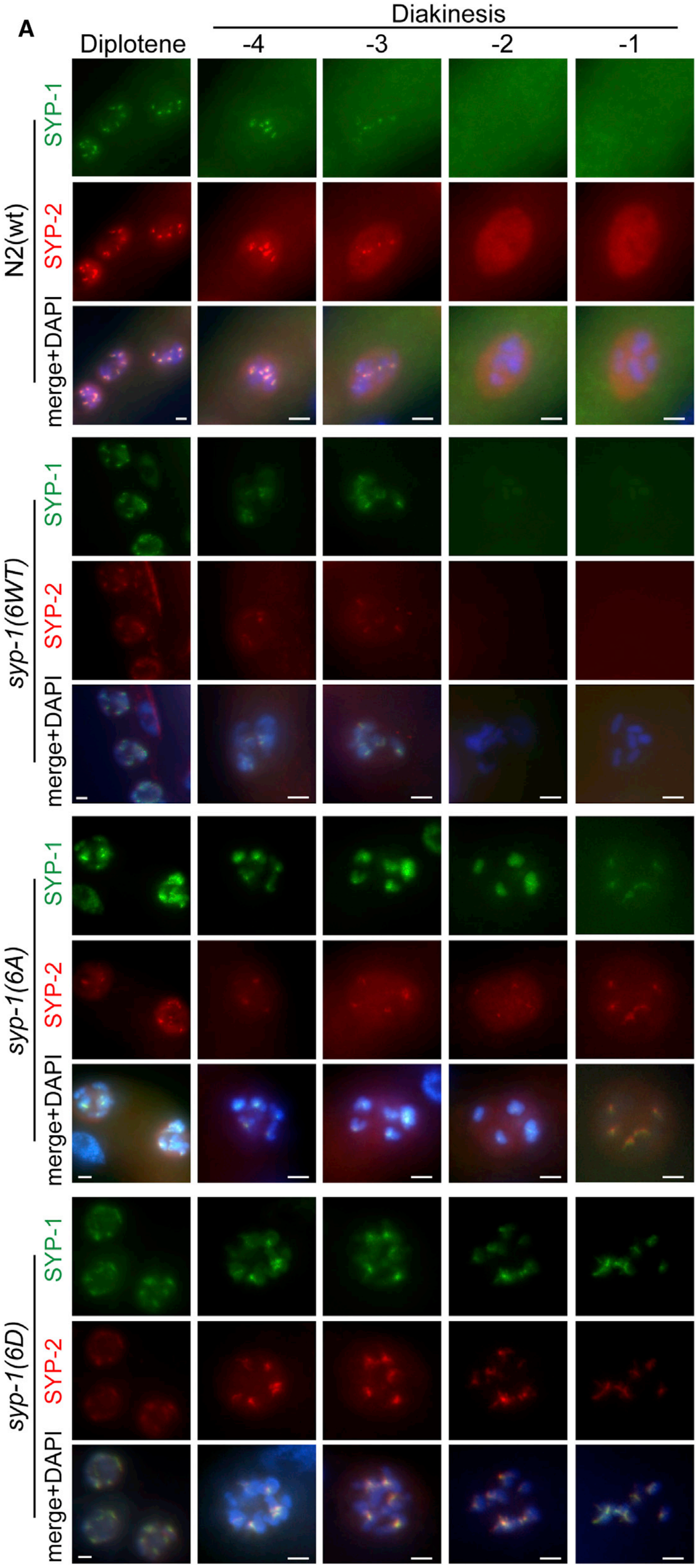

B

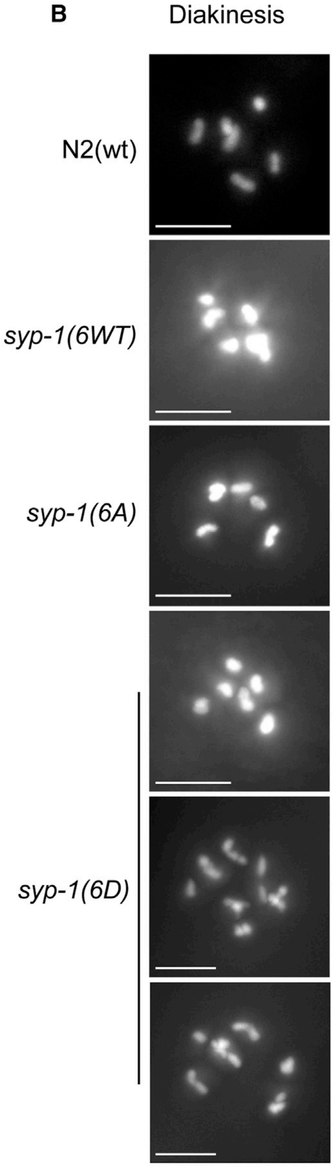

C

strain	bivalents	n
N2(wt)	6.00±0.00	32
syp-1(6WT)	6.00±0.00	42
syp-1(6A)	6.05±0.21	46
syp-1(6D)	6.14±0.41	46

(legend on next page)

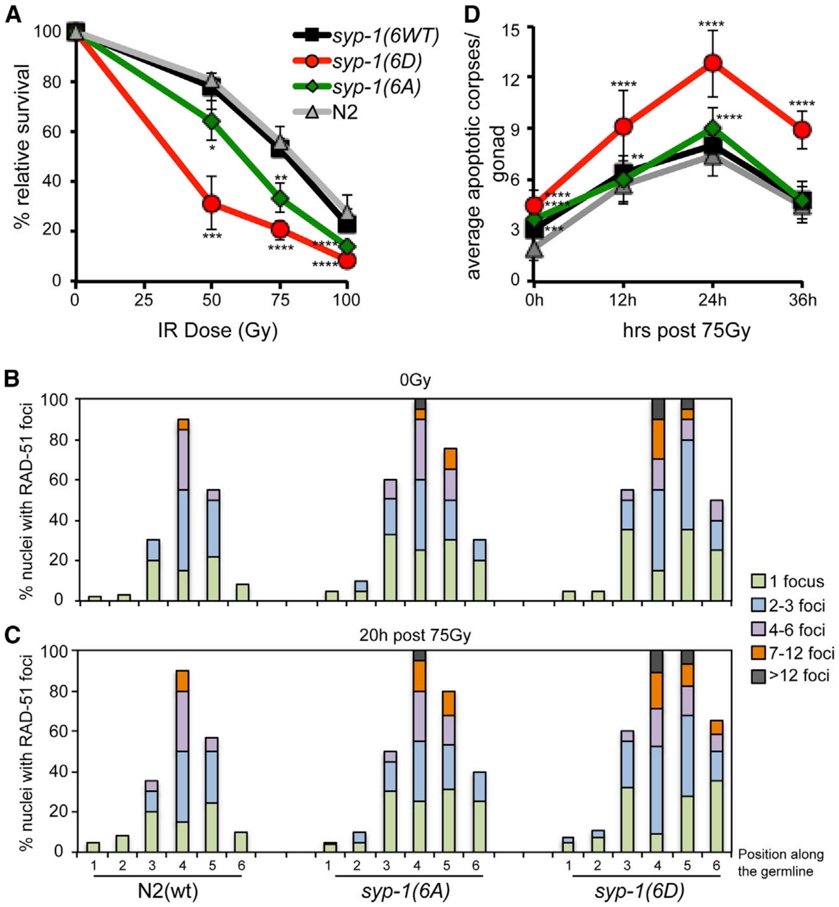

Figure 5. Defects in DNA Damage Response in the *syp-1* Phosphorylation Alleles

(A) Sensitivity of L4-stage worms from the indicated strains to different doses of IR. Relative survival of offspring is shown. Data are represented as average percentage ± SD from at least four experiments with 15 worms each. *p = 0.02, **p = 0.0015, ***p = 0.0006, ****p < 0.0001; p values for paired t test.

(B and C) Quantification of recombination marker RAD-51 foci in the indicated strains in normal conditions (B) or 20 h after 75 Gy (C). At least 15 gonads were analyzed in each condition and ten nuclei were scored in each zone (mitotic region, 1; transition zone, 2; early-mid-late pachytene regions, 3-4-5; and diplotene-diakinesis regions, 6) for at least three independent experiments.

(D) Germ cell apoptosis was measured by differential interference contrast (DIC) microscopy in animals of the indicated strains at the indicated time points after IR treatment. Data are represented as average ± SD from at least ten worms for each time point of three independent experiments. ****p < 0.0001, p value for paired t test.

syp-1 Phospho Mutant Alleles Are Lethal in a *brc-1* Background

During meiotic prophase, SPO-11-induced meiotic DSBs are repaired via HR using the homologous chromosome as a template, ensuring the formation of inter-homolog crossovers (Keeney et al., 2014). We considered the possibility that damage-induced phosphorylation of the SC could act to switch DSB repair template from the homolog to the sister chromatid to allow excess DNA lesions to be repaired without the possibility of this leading to increased inter-homolog crossovers, which could interfere with chromosome segregation at the first meiotic division. Notably, HR repair via the sister chromatid strictly depends on BRC-1, which is dispensable for inter-homolog repair (Adamo et al., 2008). Furthermore, SYP-1 co-purified with BRC-1 in the CeBCD complex (in both fractions soluble and chromatin bound), and both proteins co-localized during meiosis (Figures 2B and 6A), suggesting that BRC-1 is ideally placed to respond to phosphorylation changes in the SC.

If SYP-1 phosphorylation does indeed channel repair to the sister chromatid, then we would predict that crossing the *syp-1* mutant strains with the *brc-1*(*tm1145*) mutant would result in a synthetic phenotype. Indeed, *brc-1;syp-1*(*6A*) and *brc-1;syp-1*(*6D*) strains showed a dramatic reduction in viability corresponding to 32.9% (n = 7) and 18.3% (n = 28) viability, respectively. In contrast, the *brc-1*;*syp-1*(*6WT*) strain exhibited 99.2% (n = 19) viability (Table 1; Figure 6B), which compared with 99.4% (n = 7) viability in *brc-1* mutant worms. Furthermore, the incidence of males observed in the double *brc-1;syp-1*(*6A*) and *brc-1;syp-1*(*6D*) mutants was also elevated to 6.7% and 5.4%, respectively. Collectively, these data suggest that the damage-induced phosphorylation of SYP-1 plays a key role in the repair of exogenous and persistent meiotic DBSs. Furthermore, the genetic interaction with BRC-1 strongly suggests that SYP-1 phosphorylation or dephosphorylation controls the channelling of excessive meiotic DSBs for repair through the sister chromatid.

To understand the cause of the increased lethality of *brc-1;syp-1*(*6A*) and *brc-1;syp-1*(*6D*) strains, we examined homologous chromosome synapsis by immunostaining for the SC central region proteins. SYP-1 and SYP-2 staining in the double mutants was indistinguishable from N2(WT), both in the transition zone and throughout pachytene. Moreover, the *brc-1;syp-1*(*6A*) and *brc-1;syp-1*(*6D*) double mutants showed a similar delay in SC disassembly at diakinesis, as described above for

Figure 4. Synaptonemal Complex Disassembly in *syp-1* Alleles

(A) Representative images of diplotene region and oocytes −4 to −1 from the indicated strains' fixed germlines immunostained with synaptonemal complex protein SYP-1 and SYP-2 antibodies and counterstained with DAPI. Scale bar, 2 μm.

(B) Representative images of the diakinesis region from the indicated strains' fixed germlines stained with DAPI.

(C) Quantification of the number of DAPI-stained bodies in the diakinetic oocyte. Data are represented as average ± SD (n, number of oocytes assayed).

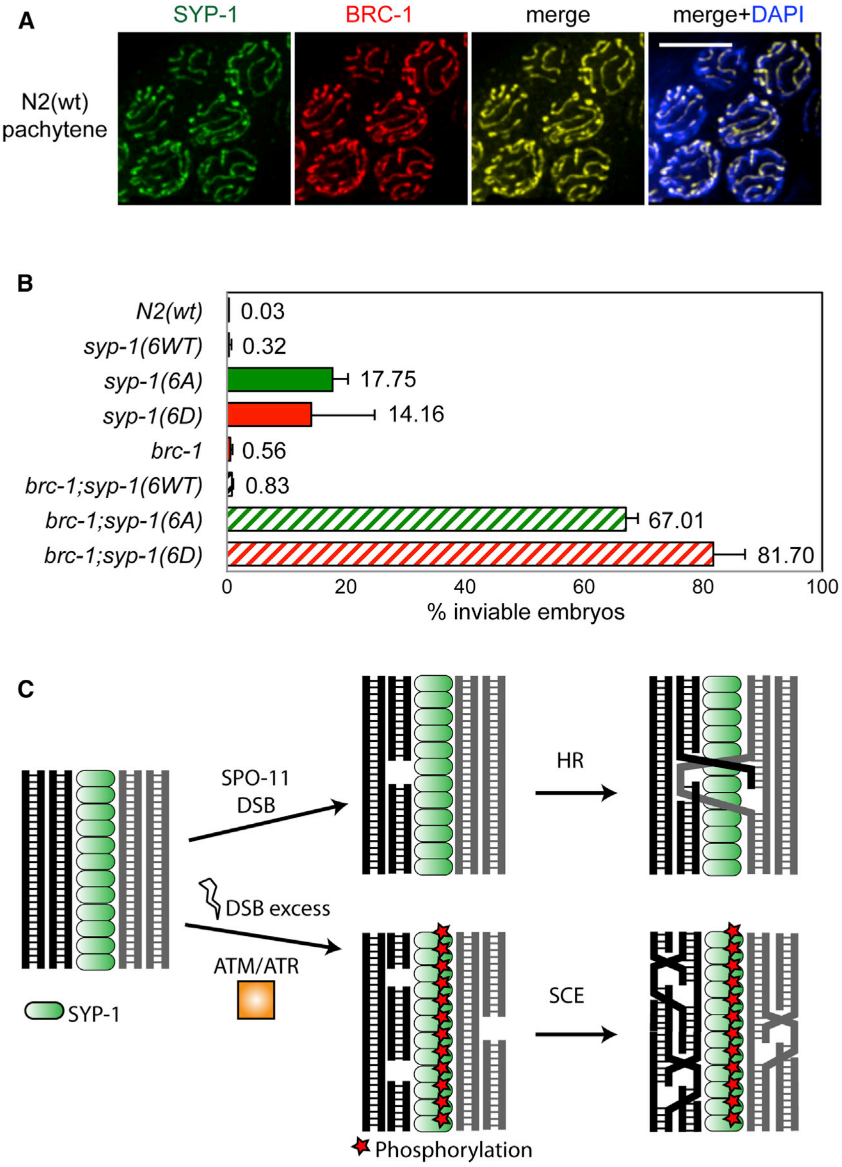

Figure 6. Embryonic Lethality of *syp-1* Phosphorylation Alleles in a *brc-1* Background

(A) Representative images of the mitotic region from N2(WT) fixed germlines immunostained with anti-BRC-1 and anti-SYP-1 antibodies and counterstained with DAPI.

(B) Percentage of embryos of the indicated genotypes that failed to complete embryogenesis. Data are represented as average percentage ± SD.

(C) Proposed model. During meiosis, SPO-11 DSBs are repaired by homologous recombination (HR) using the homolog chromatid as template (top). In a context where excessive DSBs are produced, the DNA damage checkpoint is activated and triggers phosphorylation of SC component SYP-1 to bias repair through the sister chromatid as template (bottom). For simplicity, SC is represented only with SYP-1.

the single *syp-1* mutant strains alone (Figure S7A). Notably, in the case of the double mutants, we did not notice alteration in the number of diakinetic bodies in the analyzed animals, observing 6 bivalents in all the animals analyzed. Therefore, the lethality of the double-mutant strains is not due to exacerbation of the SC phenotype.

We then monitored the repair of meiotic DSBs by quantifying RAD-51 foci. In N2(WT) and *brc-1;syp-1*(*6WT*) germlines, RAD-51 foci first appeared in the transition zone and progressively increased in number in a given nucleus, reaching a maximum in mid-pachytene and finally disappearing in late pachytene. In the case of *brc-1;syp-1*(*6A*) and *brc-1;syp-1*(*6D*) double-mutant germlines, we observed a further increase in the number of nuclei with RAD-51 foci as well as the number of foci per nuclei, particularly in the *brc-1;syp-1*(*6D*) strain (Figure S7B). Importantly a similar result was obtained when combining our *syp-1* mutant alleles with *msh-5* (Kelly et al., 2000), which is required for generating inter-homolog crossovers (Figure S7B). Since the accumulation of unrepaired DNA damage leads to apoptosis, we also scored germ cell apoptosis in the different strains. Apoptotic corpses were also significantly increased in *brc-1;syp-1*(*6D*) mutant strains when compared to the single mutants (Figure S7C), which correlates with the delayed repair of DSBs observed in these strains.

Then, we tested whether SYP-1 phosphorylation affects the timing and extent of CO designation by visualizing, in late pachytene and diplotene, ZHP-3, a protein essential for reciprocal recombination between homologous chromosomes (Bhalla et al., 2008). We observed that, while *syp-1*(*6WT*) presented with 6 foci per nucleus, both *syp-1*(*6A*) and *syp-1*(*6D*) alleles showed a significant increase in ZHP-3 foci (Figure S7D); however, we noticed a delay for ZHP-3 to become a single prominent focus on each pair of homologs, since we could only count foci in diplotene nuclei, in agreement with an abnormal resolution of DSBs in our *syp-1* mutant alleles. This is consistent with SYP-1 phosphorylation being involved in directing DSB repair through the sister chromatid. When the phospho-alleles were combined with the *brc-1* background, we observed only a modest increase in ZHP-3 foci per nucleus (Figure S7D). Taken together, these results suggest that BRC-1 is required to channel the repair of DNA breaks in the absence of proper SYP-1 phosphorylation.

DISCUSSION

DNA damage within the germline must be precisely repaired to ensure transmission of accurate genetic information to subsequent generations. While the processes that ensure high-fidelity repair of programmed meiotic DSBs to produce inter-homolog

crossovers have been extensively studied in a range of different organisms, the pathways that protect the germline from unscheduled, persistent, or excessive DNA damage remain poorly understood. Our study reveals the existence of a meiotic checkpoint in *C. elegans* that responds to excessive or persistent meiotic DSBs and functions to switch lesion repair from the homolog toward the sister chromatid. Defects in this process result in increased sensitivity to DNA damage and heightened genetic instability, highlighting the importance of this response for maintaining germline integrity.

DSBs are known to activate DNA damage checkpoint pathways, which is initiated by the two related protein kinases ATM/Tel1 and ATR/Mec1 in mammals and *S. cerevisiae*, respectively (Aguilera and García-Muse, 2013; Ciccia and Elledge, 2010). The involvement of checkpoint kinases in meiosis has been described in a range of organisms, where they have been implicated in controlling crossover formation and distribution, synapsis checkpoints, homolog pairing, and meiotic chromosome segregation (MacQueen and Hochwagen, 2011). We show here that IR-induced DSBs result in extensive Serine/Threonine glutamine (S/TQ) phosphorylation throughout the *C. elegans* meiotic germline, which is abolished by caffeine treatment (inhibits the phosphatidylinositol 3-kinase-related kinase [PIKK] family, including ATM and ATR) or the removal of both ATM and ATR checkpoint kinases (*atm-1* and *atl-1*, respectively). *C. elegans* ATM and ATR act redundantly for this meiotic checkpoint response, as strains mutated for either *atl-1* or *atm-1* retain germline phospho-S/TQ straining after DNA damage. Analysis of mutants that exhibit persistent meiotic DSBs (e.g., *rad-51*, *brc-2*, and *msh-4*/5) suggest that this meiotic checkpoint response is not limited to IR-induced DSBs but also extends to persistent meiotic DSBs that arise when normal meiotic DSB repair is delayed or compromised.

In recent years, post-translational modification by SUMOylation, N-terminal acetylation, and phosphorylation has been implicated in regulating SC dynamics (Gao and Colaiácovo, 2018). Our analysis has revealed extensive overlap between the damage-induced phospho-S/TQ staining and the SC, suggesting that key targets for this response are situated within or in close proximity to the SC and axial elements. Indeed, the mobility of SYP-1 and SYP-2 was found to be shifted in a phospho-dependent manner following IR treatment, and peptide array kinase assays using worm extracts identified a cluster of serine and threonine residues in SYP-1 that are subject to phosphorylation only in extracts from IR-treated animals. Since there are no S/T-Q sites in this region, it is likely that SYP-1 phosphorylation is mediated by kinases that are activated downstream of ATR/ATM dependence, such as CHK1 and CHK2 (Aguilera and García-Muse, 2013; Maréchal and Zou, 2013).

The SC is a dynamic structure that operates during meiosis to ensure the formation of crossovers while at the same time limiting their numbers (Colaiácovo et al., 2003; Saito and Colaiácovo, 2017). Phosphorylation of SC components has been recently reported to influence changes in SC dynamics and meiotic recombination during unperturbed meiosis (Nadarajan et al., 2017; Sato-Carlton et al., 2018). Intriguingly, a previous study reported that the SC undergoes localized disassembly during the repair of IR-induced DSBs to favor rapid repair through the sister chromatid as template (Couteau and Zetka, 2011). Furthermore, the restoration of proper reassembled SC after repair is complete requires ATM-1 (Boulton et al., 2002; Couteau et al., 2004). Since IR induces a phosphor-S/TQ (pS/TQ) signal that extends along the vast majority of the length of the SC, it is unlikely that this modification is directly responsible for the localized desynapsis of the SC, as it is not restricted to DSB sites. However, such a modification could prime the entire SC for disassembly, but this only occurs at sites that contain a break within the DNA duplex; this will be accompanied by localized chromatin modifications that are induced at sites of DSBs, which may signal SC disassembly in proximity to the DSB. Although SYP-1 is likely to be one of several targets for IR-induced phosphorylation since SYP-2 is also phosphorylated, analysis of the DDR in the *syp-1* phospho mutant alleles showed a clear impairment in dealing with the excess of IR-induced DSB, which is more dramatic in the case of the phosphomimic allele. Interestingly, the *pph-4* phosphatase mutant, which is unable to remove ATM-ATR-dependent phosphorylation marks, exhibits severe defects in sperm meiosis and oocytes with 12 univalents, suggesting that the ATR/ATM phosphorylation and dephosphorylation is important for DSB resolution (Sumiyoshi et al., 2002).

In *C. elegans*, it has been show that BRC-1 (ortholog of human BRCA1) is required exclusively for sister chromatid repair in meiosis (Adamo et al., 2008; Boulton et al., 2004). This is most clearly seen in situations where crossover formation is abrogated but meiotic DSB repair per se remains intact (such as in a *syp-1* mutant); in this context, compromising inter-sister repair by *brc-1* mutation leads to failed meiotic DSB repair and embryonic lethality (Adamo et al., 2008). We propose that the *syp-1* phospho mutant alleles act dominantly to drive meiotic DSBs toward the sister chromatid, which explains the reduced viability and increased chromosome non-dysjunction observed when combined with the *brc-1* mutant. These observations support a model (Figure 6C) in which damage-induced SYP-1 phosphorylation safeguards the germline against persistent or excessive meiotic DSBs by channelling repair to the sister chromatid.

STAR★METHODS

Detailed methods are provided in the online version of this paper and include the following:

- KEY RESOURCES TABLE
- CONTACT FOR REAGENT AND RESOURCE SHARING
- EXPERIMENTAL MODEL AND SUBJECT DETAILS
  - Strains and maintenance
  - Embryonic lethality
  - Apoptotic corpses analysis
  - MosSCI transformation
  - Worm genotyping
  - Generation of double mutants
- METHODS DETAILS
  - Constructs
  - Peptide arrays and kinase assays
  - CeBCD complex analysis
  - Immunostaining

- ○ Fluorescence microscopy
- ○ $P^{S/T-Q}$ signal quantification
- ○ SYTO12 for apoptotic corpses quantification
- ○ RAD-51 foci quantification
- ● QUANTIFICATION AND STATISTICAL ANALYSIS

SUPPLEMENTAL INFORMATION

Supplemental Information includes seven figures and one table and can be found with this article online at https://doi.org/10.1016/j.celrep.2018.12.074.

ACKNOWLEDGMENTS

We wish to thank A. Villeneuve, V. Jantsch, E. Martinez-Perez, and P. Carlton for kindly providing antibodies SYP-1 and SYP-2, SUN-1ph, HTP-1, and SYP-1ph, respectively. Boulton lab work is supported by the Francis Crick Institute, which receives its core funding from Cancer Research UK (FC0010048), the UK Medical Research Council (FC0010048), and the Wellcome Trust (FC0010048). S.J.B. is also the recipient of a European Research Council (ERC) Advanced Investigator Grant (TelMetab) and Wellcome Trust Senior Investigator and Collaborative Grants. Aguilera lab work is supported by grants from the Spanish Ministry of Economy and Competitiveness (BFU2016-75058-P), European Research Council(ERC) Advanced Investigator Grant (ERC2014 AdG669898 TARLOOP), and the European Union (FEDER). T.G.-M. was holder of postdoctoral grants from the CSICJAE-Doc and the Junta de Andalucía Excellence Program (CVI-4567).

AUTHOR CONTRIBUTIONS

T.G.-M., A.A., and S.J.B. designed the project and wrote the manuscript. T.G.-M. performed most of the experiments. U.G.-D. generated the transgenic strains. M.G.-R. helped with the double mutant with *msh-5* and counted the double-mutant apoptotic corpses. J.S.M. performed the co-localization of BRC-1 and SYP-1. J.P. performed the CeBCD complex purification and western analysis. N.O. contributed to the peptide array generation.

DECLARATION OF INTERESTS

S.J.B. is a senior group leader at the Francis Crick Institute and also SVP of Science Strategy at Artios Pharma Ltd. The work conducted in this study was supported by the FCI and was not funded or supported in any way by Artios Pharma. All other authors declare no competing interests.

Received: May 11, 2018
Revised: September 28, 2018
Accepted: December 17, 2018
Published: January 15, 2019

REFERENCES

Abraham, R.T. (2001). Cell cycle checkpoint signaling through the ATM and ATR kinases. Genes Dev. *15*, 2177–2196.

Adamo, A., Montemauri, P., Silva, N., Ward, J.D., Boulton, S.J., and La Volpe, A. (2008). BRC-1 acts in the inter-sister pathway of meiotic double-strand break repair. EMBO Rep. *9*, 287–292.

Aguilera, A., and García-Muse, T. (2013). Causes of genome instability. Annu. Rev. Genet. *47*, 1–32.

Alpi, A., Pasierbek, P., Gartner, A., and Loidl, J. (2003). Genetic and cytological characterization of the recombination protein RAD-51 in Caenorhabditis elegans. Chromosoma *112*, 6–16.

Bhalla, N., Wynne, D.J., Jantsch, V., and Dernburg, A.F. (2008). ZHP-3 acts at crossovers to couple meiotic recombination with synaptonemal complex disassembly and bivalent formation in C. elegans. PLoS Genet. *4*, e1000235.

Blasina, A., Price, B.D., Turenne, G.A., and McGowan, C.H. (1999). Caffeine inhibits the checkpoint kinase ATM. Curr. Biol. *9*, 1135–1138.

Boulton, S.J., Gartner, A., Reboul, J., Vaglio, P., Dyson, N., Hill, D.E., and Vidal, M. (2002). Combined functional genomic maps of the C. elegans DNA damage response. Science *295*, 127–131.

Boulton, S.J., Martin, J.S., Polanowska, J., Hill, D.E., Gartner, A., and Vidal, M. (2004). BRCA1/BARD1 orthologs required for DNA repair in Caenorhabditis elegans. Curr. Biol. *14*, 33–39.

Brenner, S. (1974). The genetics of Caenorhabditis elegans. Genetics *77*, 71–94.

Carballo, J.A., Johnson, A.L., Sedgwick, S.G., and Cha, R.S. (2008). Phosphorylation of the axial element protein Hop1 by Mec1/Tel1 ensures meiotic interhomolog recombination. Cell *132*, 758–770.

Ciccia, A., and Elledge, S.J. (2010). The DNA damage response: making it safe to play with knives. Mol. Cell *40*, 179–204.

Colaiácovo, M.P., MacQueen, A.J., Martinez-Perez, E., McDonald, K., Adamo, A., La Volpe, A., and Villeneuve, A.M. (2003). Synaptonemal complex assembly in C. elegans is dispensable for loading strand-exchange proteins but critical for proper completion of recombination. Dev. Cell *5*, 463–474.

Couteau, F., and Zetka, M. (2011). DNA damage during meiosis induces chromatin remodeling and synaptonemal complex disassembly. Dev. Cell *20*, 353–363.

Couteau, F., Nabeshima, K., Villeneuve, A., and Zetka, M. (2004). A component of C. elegans meiotic chromosome axes at the interface of homolog alignment, synapsis, nuclear reorganization, and recombination. Curr. Biol. *14*, 585–592.

Craig, A.L., Moser, S.C., Bailly, A.P., and Gartner, A. (2012). Methods for studying the DNA damage response in the Caenorhabdatis elegans germ line. Methods Cell Biol. *107*, 321–352.

Frøkjaer-Jensen, C., Davis, M.W., Hopkins, C.E., Newman, B.J., Thummel, J.M., Olesen, S.-P., Grunnet, M., and Jorgensen, E.M. (2008). Single-copy insertion of transgenes in Caenorhabditis elegans. Nat. Genet. *40*, 1375–1383.

Gao, J., and Colaiácovo, M.P. (2018). Zipping and Unzipping: Protein Modifications Regulating Synaptonemal Complex Dynamics. Trends Genet. *34*, 232–245.

Garcia-Muse, T., and Boulton, S.J. (2005). Distinct modes of ATR activation after replication stress and DNA double-strand breaks in Caenorhabditis elegans. EMBO J. *24*, 4345–4355.

Gray, S., Allison, R.M., Garcia, V., Goldman, A.S., and Neale, M.J. (2013). Positive regulation of meiotic DNA double-strand break formation by activation of the DNA damage checkpoint kinase Mec1(ATR). Open Biol. *3*, 130019.

Hall-Jackson, C.A., Cross, D.A., Morrice, N., and Smythe, C. (1999). ATR is a caffeine-sensitive, DNA-activated protein kinase with a substrate specificity distinct from DNA-PK. Oncogene *18*, 6707–6713.

Harper, N.C., Rillo, R., Jover-Gil, S., Assaf, Z.J., Bhalla, N., and Dernburg, A.F. (2011). Pairing centers recruit a Polo-like kinase to orchestrate meiotic chromosome dynamics in C. elegans. Dev. Cell *21*, 934–947.

Hayashi, M., Chin, G.M., and Villeneuve, A.M. (2007). C. elegans germ cells switch between distinct modes of double-strand break repair during meiotic prophase progression. PLoS Genet. *3*, e191.

Hayashi, M., Mlynarczyk-Evans, S., and Villeneuve, A.M. (2010). The synaptonemal complex shapes the crossover landscape through cooperative assembly, crossover promotion and crossover inhibition during Caenorhabditis elegans meiosis. Genetics *186*, 45–58.

Heyer, W.D., Ehmsen, K.T., and Liu, J. (2010). Regulation of homologous recombination in eukaryotes. Annu. Rev. Genet. *44*, 113–139.

Hillers, K.J., Jantsch, V., Martinez-Perez, E., and Yanowitz, J.L. (2017). Meiosis. WormBook *2017*, 1–43.

Hodgkin, J., Horvitz, H.R., and Brenner, S. (1979). Nondisjunction Mutants of the Nematode CAENORHABDITIS ELEGANS. Genetics *91*, 67–94.

Kastan, M.B., and Bartek, J. (2004). Cell-cycle checkpoints and cancer. Nature *432*, 316–323.

Keeney, S., Giroux, C.N., and Kleckner, N. (1997). Meiosis-specific DNA double-strand breaks are catalyzed by Spo11, a member of a widely conserved protein family. Cell *88*, 375–384.

Keeney, S., Lange, J., and Mohibullah, N. (2014). Self-organization of meiotic recombination initiation: general principles and molecular pathways. Annu. Rev. Genet. *48*, 187–214.

Kelly, K.O., Dernburg, A.F., Stanfield, G.M., and Villeneuve, A.M. (2000). Caenorhabditis elegans msh-5 is required for both normal and radiation-induced meiotic crossing over but not for completion of meiosis. Genetics *156*, 617–630.

Labella, S., Woglar, A., Jantsch, V., and Zetka, M. (2011). Polo kinases establish links between meiotic chromosomes and cytoskeletal forces essential for homolog pairing. Dev. Cell *21*, 948–958.

Lange, J., Pan, J., Cole, F., Thelen, M.P., Jasin, M., and Keeney, S. (2011). ATM controls meiotic double-strand-break formation. Nature *479*, 237–240.

Libuda, D.E., Uzawa, S., Meyer, B.J., and Villeneuve, A.M. (2013). Meiotic chromosome structures constrain and respond to designation of crossover sites. Nature *502*, 703–706.

Lui, D.Y., and Colaiácovo, M.P. (2013). Meiotic development in Caenorhabditis elegans. Adv. Exp. Med. Biol. *757*, 133–170.

MacQueen, A.J., and Hochwagen, A. (2011). Checkpoint mechanisms: the puppet masters of meiotic prophase. Trends Cell Biol. *21*, 393–400.

MacQueen, A.J., and Villeneuve, A.M. (2001). Nuclear reorganization and homologous chromosome pairing during meiotic prophase require C. elegans chk-2. Genes Dev. *15*, 1674–1687.

MacQueen, A.J., Colaiácovo, M.P., McDonald, K., and Villeneuve, A.M. (2002). Synapsis-dependent and -independent mechanisms stabilize homolog pairing during meiotic prophase in C. elegans. Genes Dev. *16*, 2428–2442.

Maréchal, A., and Zou, L. (2013). DNA damage sensing by the ATM and ATR kinases. Cold Spring Harb. Perspect. Biol. *5*, a012716.

Martin, J.S., Winkelmann, N., Petalcorin, M.I., McIlwraith, M.J., and Boulton, S.J. (2005). RAD-51-dependent and -independent roles of a Caenorhabditis elegans BRCA2-related protein during DNA double-strand break repair. Mol. Cell. Biol. *25*, 3127–3139.

Martinez-Perez, E., and Villeneuve, A.M. (2005). HTP-1-dependent constraints coordinate homolog pairing and synapsis and promote chiasma formation during C. elegans meiosis. Genes Dev. *19*, 2727–2743.

Martinez-Perez, E., Schvarzstein, M., Barroso, C., Lightfoot, J., Dernburg, A.F., and Villeneuve, A.M. (2008). Crossovers trigger a remodeling of meiotic chromosome axis composition that is linked to two-step loss of sister chromatid cohesion. Genes Dev. *22*, 2886–2901.

Nadarajan, S., Lambert, T.J., Altendorfer, E., Gao, J., Blower, M.D., Waters, J.C., and Colaiácovo, M.P. (2017). Polo-like kinase-dependent phosphorylation of the synaptonemal complex protein SYP-4 regulates double-strand break formation through a negative feedback loop. eLife *6*, e23437.

Parusel, C.T., Kritikou, E.A., Hengartner, M.O., Krek, W., and Gotta, M. (2006). URI-1 is required for DNA stability in C. elegans. Development *133*, 621–629.

Pasierbek, P., Jantsch, M., Melcher, M., Schleiffer, A., Schweizer, D., and Loidl, J. (2001). A Caenorhabditis elegans cohesion protein with functions in meiotic chromosome pairing and disjunction. Genes Dev. *15*, 1349–1360.

Penkner, A.M., Fridkin, A., Gloggnitzer, J., Baudrimont, A., Machacek, T., Woglar, A., Csaszar, E., Pasierbek, P., Ammerer, G., Gruenbaum, Y., and Jantsch, V. (2009). Meiotic chromosome homology search involves modifications of the nuclear envelope protein Matefin/SUN-1. Cell *139*, 920–933.

Phillips, C.M., and Dernburg, A.F. (2006). A family of zinc-finger proteins is required for chromosome-specific pairing and synapsis during meiosis in C. elegans. Dev. Cell *11*, 817–829.

Phillips, C.M., Wong, C., Bhalla, N., Carlton, P.M., Weiser, P., Meneely, P.M., and Dernburg, A.F. (2005). HIM-8 binds to the X chromosome pairing center and mediates chromosome-specific meiotic synapsis. Cell *123*, 1051–1063.

Polanowska, J., Martin, J.S., Garcia-Muse, T., Petalcorin, M.I.R., and Boulton, S.J. (2006). A conserved pathway to activate BRCA1-dependent ubiquitylation at DNA damage sites. EMBO J. *25*, 2178–2188.

Saito, T.T., and Colaiácovo, M.P. (2017). Regulation of Crossover Frequency and Distribution during Meiotic Recombination. Cold Spring Harb. Symp. Quant. Biol. *82*, 223–234.

Sarkaria, J.N., Busby, E.C., Tibbetts, R.S., Roos, P., Taya, Y., Karnitz, L.M., and Abraham, R.T. (1999). Inhibition of ATM and ATR kinase activities by the radiosensitizing agent, caffeine. Cancer Res. *59*, 4375–4382.

Sato-Carlton, A., Nakamura-Tabuchi, C., Chartrand, S.K., Uchino, T., and Carlton, P.M. (2018). Phosphorylation of the synaptonemal complex protein SYP-1 promotes meiotic chromosome segregation. J. Cell Biol. *217*, 555–570.

Schild-Prüfert, K., Saito, T.T., Smolikov, S., Gu, Y., Hincapie, M., Hill, D.E., Vidal, M., McDonald, K., and Colaiácovo, M.P. (2011). Organization of the synaptonemal complex during meiosis in Caenorhabditis elegans. Genetics *189*, 411–421.

Schindelin, J., Arganda-Carreras, I., Frise, E., Kaynig, V., Longair, M., Pietzsch, T., Preibisch, S., Rueden, C., Saalfeld, S., Schmid, B., et al. (2012). Fiji: an open-source platform for biological-image analysis. Nat. Methods *9*, 676–682.

Shiloh, Y. (2001). ATM and ATR: networking cellular responses to DNA damage. Curr. Opin. Genet. Dev. *11*, 71–77.

Siegel, J.J., and Amon, A. (2012). New insights into the troubles of aneuploidy. Annu. Rev. Cell Dev. Biol. *28*, 189–214.

Smolikov, S., Eizinger, A., Schild-Prufert, K., Hurlburt, A., McDonald, K., Engebrecht, J., Villeneuve, A.M., and Colaiácovo, M.P. (2007). SYP-3 restricts synaptonemal complex assembly to bridge paired chromosome axes during meiosis in Caenorhabditis elegans. Genetics *176*, 2015–2025.

Smolikov, S., Schild-Prüfert, K., and Colaiácovo, M.P. (2009). A yeast two-hybrid screen for SYP-3 interactors identifies SYP-4, a component required for synaptonemal complex assembly and chiasma formation in Caenorhabditis elegans meiosis. PLoS Genet. *5*, e1000669.

Stiernagle, T. (2006). Maintenance of C. elegans. WormBook, 1–11.

Sumiyoshi, E., Sugimoto, A., and Yamamoto, M. (2002). Protein phosphatase 4 is required for centrosome maturation in mitosis and sperm meiosis in C. elegans. J. Cell Sci. *115*, 1403–1410.

Villeneuve, A.M. (1994). A cis-acting locus that promotes crossing over between X chromosomes in Caenorhabditis elegans. Genetics *136*, 887–902.

Zetka, M.C., Kawasaki, I., Strome, S., and Müller, F. (1999). Synapsis and chiasma formation in Caenorhabditis elegans require HIM-3, a meiotic chromosome core component that functions in chromosome segregation. Genes Dev. *13*, 2258–2270.

STAR★METHODS

KEY RESOURCES TABLE

REAGENT or RESOURCE	SOURCE	IDENTIFIER
Antibodies		
Rabbit Phospho-(Ser/Thr) ATM-ATR Substrate Antibody	Cell Signaling	2851
Guinea Pig SYP-1 Antibody	The A. Villenueve lab	MacQueen et al., 2002
Rabbit RAD-51 Antibody	Novus Biologicals	NB100-148
Rabbit SYP-2 Antibody	The A. Villenueve lab	Colaiácovo et al., 2003
Rabbit HIM-8 Antibody	Novus Biologicals	41980002
Rabbit BRC-1 Antibody	The S. Boulton lab	N/A
Alexa Fluor Goat anti-Guinea Pig::488	Life Technologies	A11073
Alexa Fluor Goat anti-Rabbit::568	Life Technologies	A11011
Guinea pig SUN-1-ph Antibody	The V. Jantsch lab	Penkner et al., 2009
Rabbit HTP-1 Antibody	The E. Perez-Martinez lab.	Martinez-Perez et al., 2008
Rabbit SYP-1-ph Antibody	The P. Carlton lab.	Sato-Carlton et al., 2018
Guinea Pig ZHP-3	The S. Boulton lab	Bhalla et al., 2008
Bacterial Strains		
Escherichia coli DH5a chemically competent cells	N/A	N/A
One Shot ccdB Survival 2T1 chemically competent cells	Invitrogen	# A10460
Chemicals, Peptides, and Recombinant Proteins		
Alkaline Phosphatase	Roche	713 023
Caffeine	Sigma	C0750
Vectashield	Vector Laboratories	H-1000
4,6-Diamidino-2-phenylindole dihydrochloride (DAPI)	Sigma	D9542
Peptide array (19-mer peptides on cellulose membrane)	N/A	N/A
Phusion High-Fidelity DNA polymerase	NEB	M0530S
MyTaq	BIOLINE	BIO-21107
MaeIII	Roche	10822230001
BstAPI	NEB	R0654S
Hpy188	NEB	R0617S
Experimental Models: Organisms/Strains		
C. elegans: Strain N2: wild-type Bristol	CGC	WB Strain: N2
C. elegans: Strain AV307: *syp*-1(me17) V/nT1[*unc*-?(n754) *let*-? qIs50] (IV;V)	CGC	WB Strain: AV307; WormBase: WBVar00088867
C. elegans: Strain AV276: *syp*-2(ok307)V/nT1[*unc*-?(n754) *let*-?(m435)] (IV;V)	CGC	WB Strain: AV276; WormBase: WBVar00091605
C. elegans: Strain AV271: *him*-3(me80)	CGC	WB Strain: AV271; WormBase: WBVar00088878
C. elegans: Strain VC666: *rec*-8(ok978) IV/nT1[qIs51] (IV;V)	CGC	WB Strain: VC666; WormBase: WBVar00092249
C. elegans: Strain VC381: *atm*-1(gk186) I	CGC	WB Strain: VC381; WormBase: WBVar00145593
C. elegans: Strain DW101: *atl*-1(tm853) IV/ nT1[qIs50] (IV;V)	CGC	WB Strain: DW101; WormBase: WBVar00249879
C. elegans: Strain GIN105: *atm*-1(gk186) I; *atl*-1(tm853) IV/ nT1 [qIs50] (IV;V)	This study	N/A

(*Continued on next page*)

Continued

REAGENT or RESOURCE	SOURCE	IDENTIFIER
C. elegans: Strain DW104: *brc*-2(tm1086)III/ hT2[*bli*-4(e937) *let*-?(q748)qls48] (I;III)	CGC	WB Strain: DW104; WormBase: WBVar00250104
C. elegans: Strain AV115: *msh*-5(me23)IV/ nT1[*unc*-?(n754) let-?(m435)] (IV;V)	CGC	WB Strain: AV115; WormBase: WBVar00088870
C. elegans: Strain AV112: *mre*-11(ok179) IV/nT1[*unc*-?(n754) let-?] (IV;V)	CGC	WB Strain: AV112; WormBase: WBVar00091492
C. elegans: Strain AV146: *chk*-2(me64)*rol*-9(sc148)/ *unc*-51(e369) *rol*-9(sc148) (V)	CGC	WB Strain: AV146; WormBase: WBVar00088876
C. elegans: Strain AV106: *spo*-11(ok79)IV/ nT1[*unc*-?(n754)let-?] (IV;V)	CGC	WB Strain: AV106; WormBase: WBVar00091464
C. elegans: Strain TG9: *dpy*-13(e184)*rad*-51(lg8701) IV/ nT1 [*let*-?(m435)] (IV;V)	CGC	WB Strain: G9; WormBase: WBVar00088499
C. elegans: Strain DW103: *brd*-1(dw1) III	CGC	WB Strain: DW103; WormBase: WBVar00142874
C. elegans: Strain DW102: *brc*-1(tm1145) III	CGC	WB Strain: DW102; WormBase: WBVar00250161
C. elegans: Strain EG4322: *ttTi5605; unc-119(ed9)* (II;III)	CGC	WB Strain: EG4322; WormBase: WBVar00254893
C. elegans: Strain DWIs3: [$P_{brd\text{-}1}$*brd-1*::tag]	Polanowska et al., 2006	DWIs3
C. elegans: Strain GIN107: [$P_{syp\text{-}1}$*syp-1 6WT+ unc-119(+)*] ; *syp*-1(me17) (II;V)	This study	N/A
C. elegans: Strain GIN108: [$P_{syp\text{-}1}$*syp-1 6A + unc-119(+)*] ; *syp*-1(me17) (II;V)	This study	N/A
C. elegans: Strain GIN109: [$P_{syp\text{-}1}$*syp-1 6D + unc-119(+)*] ; *syp*-1(me17) (II;V)	This study	N/A
C. elegans: Strain GIN113: [$P_{syp\text{-}1}$*syp-1 6WT+ unc-119(+)*] ; *brc*-1 (tm1145); *syp*-1(me17) (II;III;V)	This study	N/A
C. elegans: Strain GIN115: [$P_{syp\text{-}1}$*syp-1 6A+ unc-119(+)*] ; *brc*-1 (tm1145); *syp-1*(me17) (II;III; V)	This study	N/A
C. elegans: Strain GIN117: [$P_{syp\text{-}1}$*syp-1 6D+ unc-119(+)*] ; *brc*-1 (tm1145); *syp-1*(me17) (II;IV;V)	This study	N/A
C. elegans: Strain GIN115: [$P_{syp\text{-}1}$*syp-1 6A+ unc-119(+)*] ; *msh-5*(me23); *syp-1*(me17) (II;IV;V)/ nT1[*unc*-?(n754)let-?] (IV;V)	This study	N/A
C. elegans: Strain GIN117: [$P_{syp\text{-}1}$*syp-1 6D+ unc-119(+)*] ; *msh-5* (me23); *syp-1*(me17) (II;IV;V)/ nT1[*unc*-?(n754)let-?] (IV;V)	This study	N/A
C. elegans: Strain AV115: *msh-5*(me23) (IV)/ nT1[*unc*-?(n754) let-?] (IV;V)	Kelly et al., 2000	WB Strain: AV115; WormBase: WBVar00088870
Oligonucleotides		
Primers for cloning *syp-1* phospho-alleles, see Table S1	This study	N/A
Primers for sequencing *syp-1* phospho-alleles integration, see Table S1	This study	N/A
Primers for genotyping *syp-1* phospho-alleles integration, see Table S1	This study	N/A
Primers for genotyping *syp-1*(me17) mutant allele, see Table S1	This study	N/A
Primers for genotyping *brc-1*(tm1145) mutant allele, see Table S1	This study	N/A
Primers for genotyping *msh-5*(me23) mutant allele, see Table S1	This study	N/A
Recombinant DNA		
pDONOR 221	Invitrogen	#12536-017
pCFJ151	Frøkjaer-Jensen et al., 2008	N/A
pJLH3.1 (Pglh-2::transposase)	Frøkjaer-Jensen et al., 2008	N/A

(*Continued on next page*)

Continued		
REAGENT or RESOURCE	SOURCE	IDENTIFIER
pGH8 (Prab-3::mCherry)	Frøkjaer-Jensen et al., 2008	N/A
pCFJ90 (Pmyo-2::mCherry)	Frøkjaer-Jensen et al., 2008	N/A
pCFJ104 (Pmyo-3::mCherry)	Frøkjaer-Jensen et al., 2008	N/A
pMOS-syp-1(6WT)	This study	N/A
pMOS-syp-1(6A)	This study	N/A
pMOS-syp-1(6D)	This study	N/A
Software and Algorithms		
ImageJ (FIJI)	https://imagej.net/Welcome	Schindelin et al., 2012
Leica Application Suite Advanced Fluorescence (LAS-AF)	Leica	N/A
Nikon Instruments Software (NIS)	Nikon	N/A
Other		
Gateway® Vector Conversion Reagent System	Invitrogen	#11828-029

CONTACT FOR REAGENT AND RESOURCE SHARING

Further information and requests for reagents should be directed to and will be fulfilled by the Lead Contact, Tatiana Garcia-Muse (tatiana.muse@cabimer.es).

EXPERIMENTAL MODEL AND SUBJECT DETAILS

Strains and maintenance

Standard methods were used for the maintenance and manipulation of *C. elegans* strains (Brenner, 1974; Stiernagle, 2006). Nematode strains were provided by the *Caenorhabditis* Genetics Center, which is funded by the NIH National Center for Research Resources. The strains with transgenic *syp-1* alleles were generated using MosSCI (Frøkjaer-Jensen et al., 2008) by microinjection into *unc-119* segregants from strain EG4322 [ttTi5605 (II); *unc-119*(ed3) (III)] see below. All strains used in this study are listed in the Key Resources Table (KRT).

Embryonic lethality

Embryonic lethality was scored by comparing the number of eggs that hatch to produce viable progeny versus the total number of eggs laid. Briefly L4 hermaphrodites grown at 20°C were individually plated. The animals were transferred to new plates once every 24 hours until the egg laying stopped. Eggs laid were immediately counted. When each brood reached adulthood, the total number of live animals per brood was counted and checked against the egg count to give the total brood size and an estimate of the embryonic lethality frequency. The number of larval arrested and male progeny animals was also noted. In each experiment a minimum of three animals were analyzed and the total number of single hermaphrodites for each stain is indicated in Table 1.

For brood analysis after irradiation, post-L4 animals were exposed to the indicated Gy doses of γ-ray from BioBeam8000. After 24-hour five post-irradiation P0 worms were plated to lay eggs between 12 to 14 hr. 24 h later the number of hatched F1 larvae and dead embryos were counted (Craig et al., 2012). At least three plates were counted for each strain and condition, and the experiment was repeated four times.

Apoptotic corpses analysis

For apoptotic corpses (AP) analysis after irradiation, 24 hours post-L4 animals were exposed to 75Gy of γ-ray from BioBeam8000. After the indicated times post-irradiation worms were transferred to slides with agarose pad to observed under the microscope and APs were determined by DIC optics (Craig et al., 2012). At least 15 worms were counted for each strain and condition, and the experiment was repeated three times.

MosSCI transformation

MosSCI transformation was performed based on the protocol described in Frøkjaer-Jensen et al., (2008) (https://sites.google.com/site/jorgensenmosscі/). The Mos1 insertion strains EG4322 was used for injection. Injection mixes contained pJL43.1 (50 ng/ml), pGH8 (10 ng/ml), pCFJ104 (5 ng/ml), pCFJ90 (2 ng/ml) and the respective expression clone (50 ng/ml) in 20 mM potassium phosphate and 3 mM potassium citrate (pH 7.5). The resulting transformants (moving worms with fluorescence) were transferred to new plates until candidates (moving worms without fluorescence) arise, which were isolated and genotyped.

Worm genotyping

The resulting transformants were check by single worm PCR using MyTaq DNA-polymerase. gDNA was obtained by single worm lysis and used in nested PCRs to check for integration (primers Cbunc-119 E2/ tti5606 E3 and Cbunc-119 I2/ tti5606 I3 for the external and internal PCR respectively, are listed in Table S1) and homocigosis (tti5606 E1/ tti5606 E4 and tti5606 I1/ tti5606 I4 for the external and internal PCR respectively, are listed in KRT).

Generation of double mutants

Homozygous transgenic worms were then crossed with *syp-1(me17)* point mutant. The final strains were check by single worm PCR using MyTaq DNA-polymerase. The presence of *syp-1(me17)* allele was determined by taking advance of the FR the mutation generates. A fragment of the gene amplified with nested PCR (syp-1 primers for the external and internal PCR respectively, are listed in Table S1) and then digested using MaeIII and BstAPI restriction enzymes.

Final phospho-alleles strains were crossed with *brc-1(tm1145)* deletion mutant. The double mutant strains were check by single worm PCR using MyTaq DNA-polymerase. The presence of *brc-1(tm1145)* deletion allele was determined by nested PCR (primers brc-1 E1/ brc-1 E2 and brc-1 I1/ brc-1 I2 for the external and internal PCR respectively, are listed in Table S1).

Final phospho-alleles strains were crossed with *msh-5(me23)* mutant. The double mutant strains were check by single worm PCR using MyTaq DNA-polymerase. The presence of *msh-5(me23)* allele was determined by taking advance of the FR the mutation generates. A fragment of the gene was amplified with nested PCR (primers msh-5I1/ msh-5I2 and msh-5I3/ msh-5I3 for the external and internal PCR respectively, are listed in Table S1) and then digested using Hpy188I restriction enzyme.

METHODS DETAILS

Constructs

Targeting transgenes containing phospho mutant *syp-1*(6A) and phospho-mimetic *syp-1*(6D) were constructed by two PCR step with oligonucleotides containing the specific sequence (6A or 6D respectively) and cloned into the Invitrogen Gateway entry vector p221 for sequencing (primers listed in Table S1). Targeting transgene containing the wild-type *syp-1*(6WT) was constructed identically but with one step PCR. Microinjection plasmids carrying the *syp-1* alleles were obtained using the Invitrogen Gateway System (cat. no. 12537-023) with a pCFJ151 (Frøkjaer-Jensen et al., 2008) modified to contain the gateway cassette with kit (cat. no. 12537-023). All cloning PCR amplifications were done with a high-fidelity Phusion polymerase.

Peptide arrays and kinase assays

For the peptide array studies, 18-mer peptides were made by solid-phase synthesis and purified by high-performance liquid chromatography, and their sequences were verified by mass spectroscopy. The 18-mer peptides peptides juxtaposed by three amino acids until scanning the complete SYP-1 protein. All peptides contained an N-terminal biotin group with an aminohexanoic spacer to be spotted onto cellulose membrane. The membrane was activated by soaking in methanol for 2 min and washed twice with kinase buffer supplemented with 3% BSA. *In vitro* phosphorylation was performed by incubating the membrane in 5 mL of kinase buffer supplemented with N2 worm extracts (protein concentration of 10 mg/ mL) and 100 μCi of [32P] γ-ATP. After adding stop buffer, the membrane was washed sequentially in 1 M NaCl, then 1% SDS, and finally 0.5% phosphoric acid solution. After washing in 96% ethanol, the membrane was dried and exposed to autoradiography film.

CeBCD complex analysis

Purification of CeBCD complex by tandem immunoaffinity was performed as described in (Polanowska et al., 2006). Briefly, the *dwIs3* transgenic line was grown to high density in a 60L BioFlo5000 fermenter. Then untreated and irradiated (12h post 75Gy) worms were harvested using a Cepa continuous centrifuge and lysed in CSK buffer. Soluble supernatant fraction (S) was collected by centrifugation and the chromatin bound fraction (C) was extracted from the pellet by micrococal nuclease (Roche) treatment (3U/μl). Tandem immunoaffinity purification of the native CeBCD complex was performed using MAb12CA5 (HA) then MAb9E10 (Myc) antibody affinity columns and then eluted from the final column by cleavage of the TAG using recombinant TEV protease (Invitrogen).

Western blotting was performed with antibodies to SYP-1 and SYP-2 (1:500) and BRD-1 (1:200). To assess the nature of the mobility shift of SYP-1 and SYP-2, the protein samples were treated with alkaline phosphatase.

Immunostaining

For all the antibodies used in this study worms were treated as described (Martin et al., 2005). One day post-L4 adult gonads were dissected in PBS on poly-lysine slides, fixed for 30 minutes in 4% paraformaldehyde and replaced for 5 minutes in TBSBTx (TBSB + 0.1% TX100). The slides were washed twice for 10 minutes and one more for 30 minutes with TBSB (TBS + 0.5% BSA). They were incubated overnight at 4°C with the antibody (listed in KRT). Dilutions used: rabbit α-$P^{S/T-Q}$ (1:1000), rabbit α-RAD-51 (1:10000), guinea pig α-SYP-1 (1:10000), rabbit α-SYP-2 (1:10000), rabbit α-HIM-8 (1:200), rabbit α-BRC-1 (1:200), rabbit α-HTP-1 (1:400), guinea pig α-SUN-1ph (1:700), and guinea pig α-ZHP-3 (1:250) in TBSB. Next day gonads were rinse and then washed 3 times in TBSB, each for 20 minutes at RT, and incubated for 2 hours with the secondary antibody in TBSB (αRABBIT 1:5000, αGUINEA

PIG 1:5000), but for SUN-1p and SYP-1ph (αRABBIT 1:500, αGUINEA PIG 1:500, respectively). Gonads were rinse and then washed three times for 20 minutes in TBSB and mounted with 10 μL Vectashield (with 1 μg/ml DAPI) per sample for further analysis.

Fluorescence microscopy

Three-dimensional datasets were computationally deconvolved, and regions of interest then projected into one dimension.

Leica DM6000B was used to examine the germlines with 40X HCXPL-APO/1.25 OIL, 63X HCXPL-APO/1.40 OIL or 100X HCXPL-APO/1.40 OIL lens, and images captured using Leica LAS-AF computer software.

Nikon SMZ-645 was used to examine the germlines with 40X CFI PLAN DLL/1.25 OIL, 60X PL-APO/1.45 OIL or 100X PL-APO/1.45 OIL lens, and images captured using Nikon NIS computer software.

$P^{S/T\text{-}Q}$ signal quantification

The $P^{S/T\text{-}Q}$ intensity date for the different strains was obtained from tiff files using the ImageJ software (Fiji, Schindelin et al., 2012). For each tiff circles of the same size containing the DAPI signal established the nuclei area, then the Raw Integrated Densite data was obtained for the appropriated channel ($P^{S/T\text{-}Q}$). The graph shows the average ± SD from a minimum of 20-30 nuclei/germline of each strain.

SYTO12 for apoptotic corpses quantification

For apoptotic corpses (AP) analysis in the double mutants, worms were incubated in the staining solution (33mM aqueous solution of SYTO 12 for approximately 4h at RT). Then worms were transferred to new dishes and incubated in the dark for approximately for 45 minutes at 20°C. Finally worms were put in slides with agarose pad to observe under the microscope. At least 10 worms were counted for each strain and condition, and the experiment was repeated as least three times.

RAD-51 foci quantification

Leica DM6000B and Nikon inverted microscope was used to examine the germlines with 63X HCXPL-APO/1.40 OIL lens. Ten nuclei were counted for each region from at least 30 animals along independent experiments. Data shows the % of nuclei of the different categories based in the number of foci/nuclei.

QUANTIFICATION AND STATISTICAL ANALYSIS

Statistical significance was determined with a Student's t test using PRISM software (Graphpad Software Inc.). Statistically significant differences were labeled with one, two, three or four asterisks if *p = 0.02, **p = 0.0015, ***p = 0.0006, **** p < 0.0001, respectively. Specific replicate numbers (n) for each experiment can be found in the corresponding figure legends. In all figures, means are plotted and standard deviation (SD) is represented as error bars.

Cell Reports, Volume *26*

Supplemental Information

A Meiotic Checkpoint Alters Repair Partner Bias

to Permit Inter-sister Repair of Persistent DSBs

Tatiana Garcia-Muse, U. Galindo-Diaz, M. Garcia-Rubio, J.S. Martin, J. Polanowska, N. O'Reilly, A. Aguilera, and Simon J. Boulton

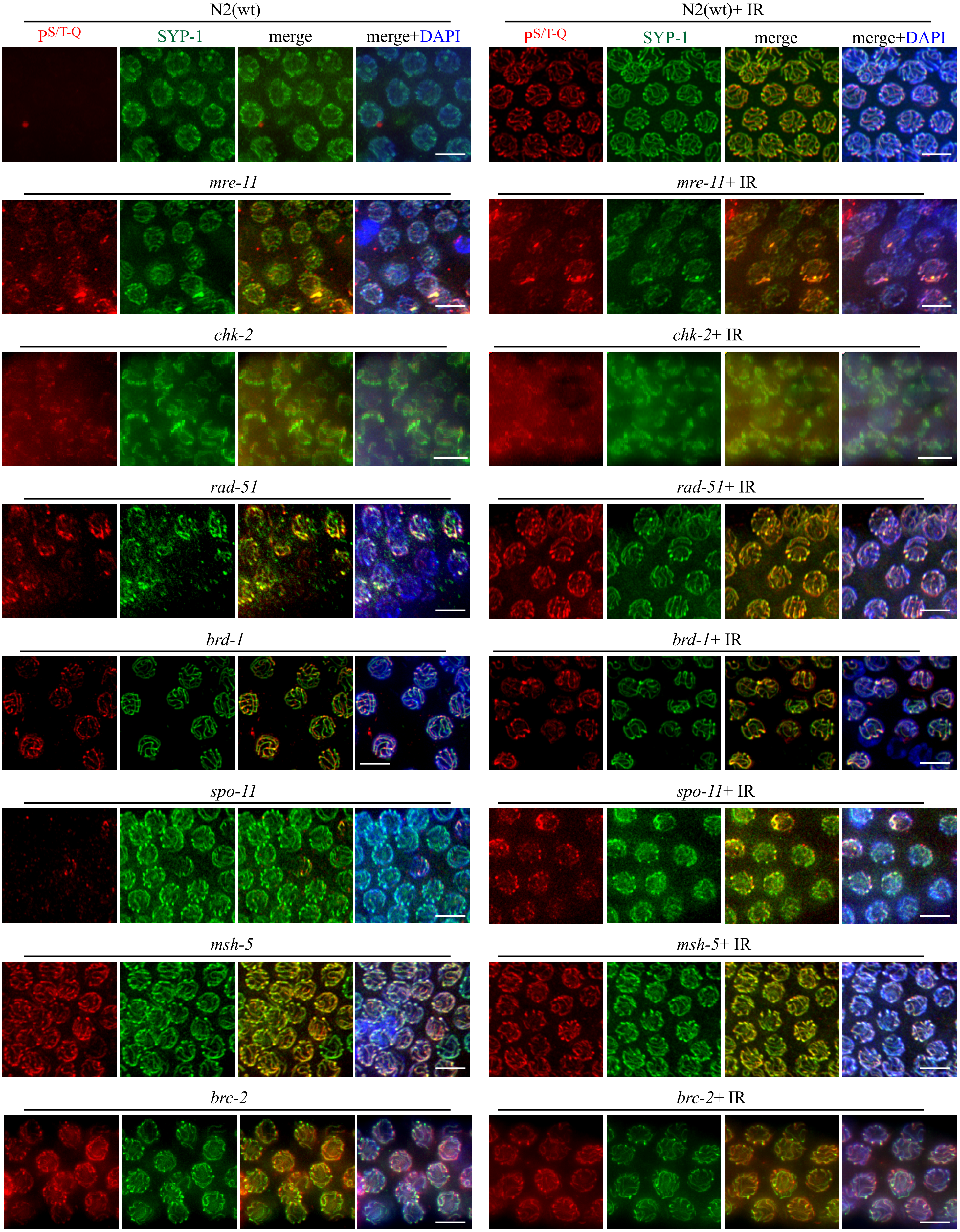

Figure S1. $P^{S/T\text{-}Q}$ phosphorylation in DNA repair and DNA damage response mutants, Related to Figure 1. Representative images of the pachytene region from the indicated strains fixed germlines immunostained with anti-$P^{S/T\text{-}Q}$ and Synaptonemal Complex protein SYP-1 antibodies and counterstained with DAPI without DNA damage or 1 hour post 75Gy. Scale bar 5µm.

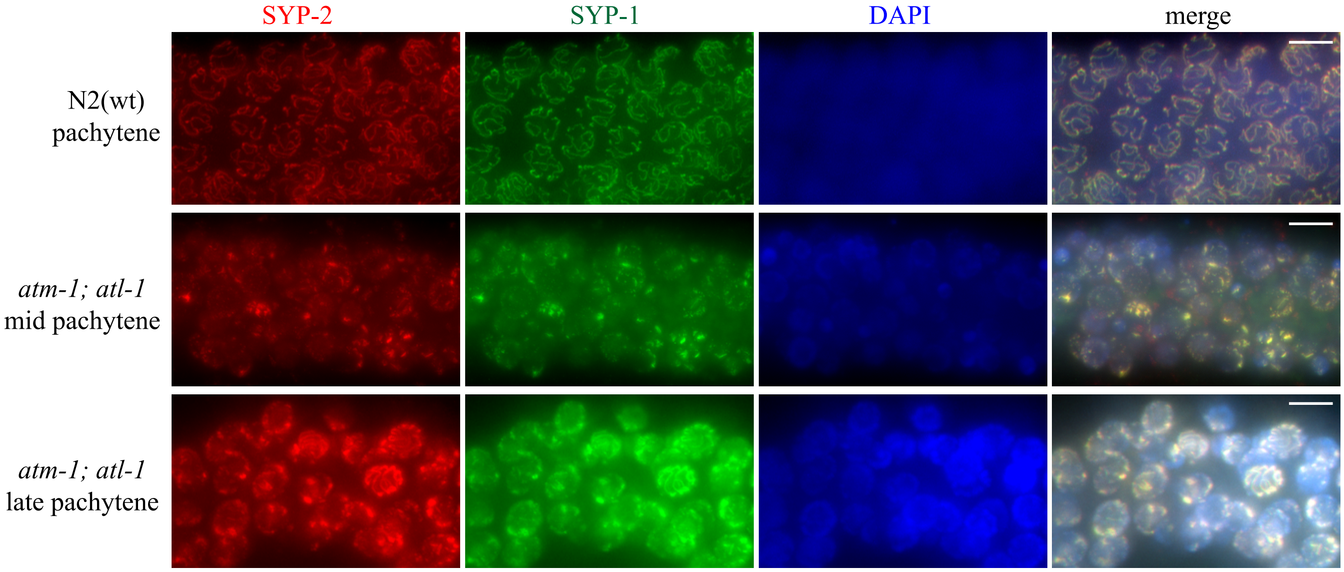

Figure S2. Synaptonelmal Complex is not well assembled in the *atm-1; atl-1* mutant, Related to Figure 1. Representative images of the pachytene region from the indicated strains fixed germlines immunostained with Synaptonemal Complex proteins SYP-1 and SYP-2 antibodies counterstained with DAPI. Scale bar 5μm.

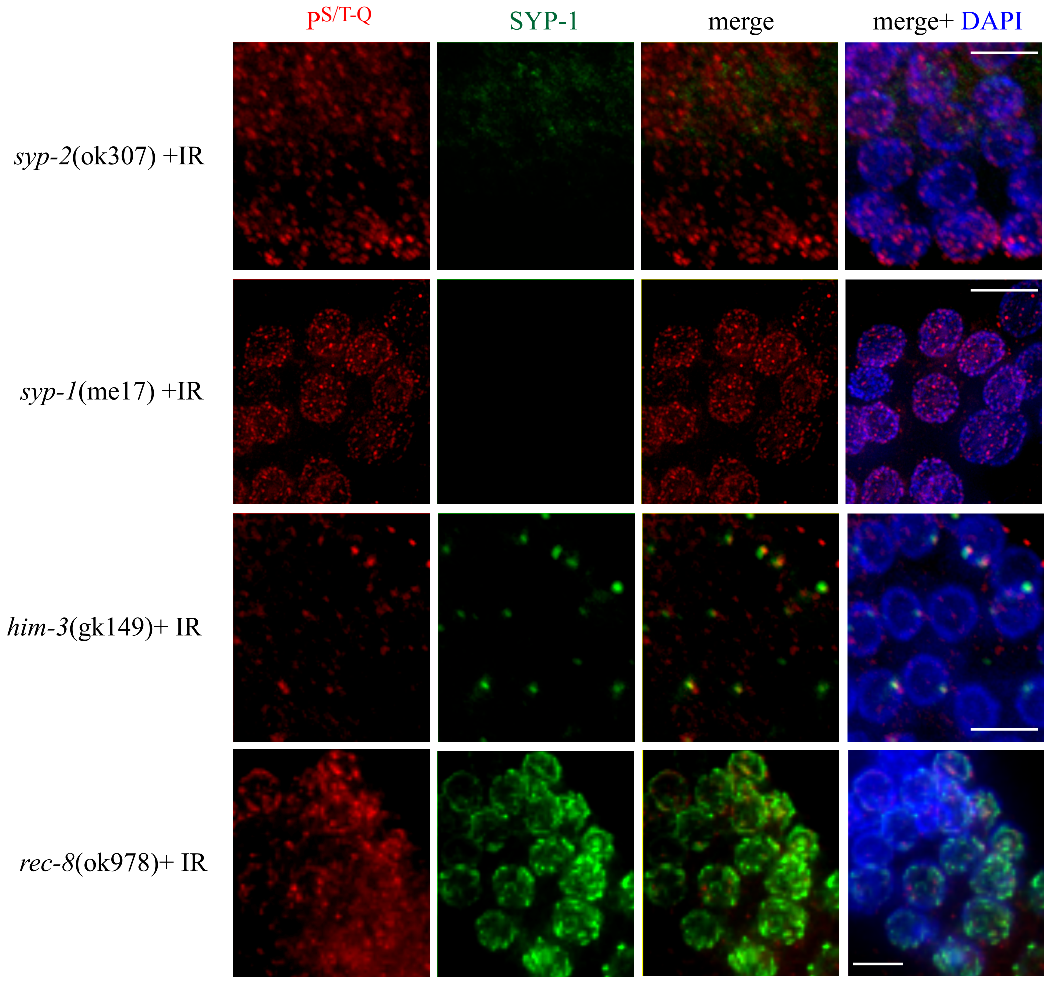

Figure S3. Single mutant screening for the target of the ATM/ATR phosphorylation in response to DNA damage, Related to Figure 2. Representative images of the pachytene region from the indicated strains fixed germlines immunostained with anti-$P^{S/T\text{-}Q}$ and Synaptonemal Complex protein SYP-1 antibodies counterstained with DAPI 1 hour post 75Gy. Scale bar 5µm.

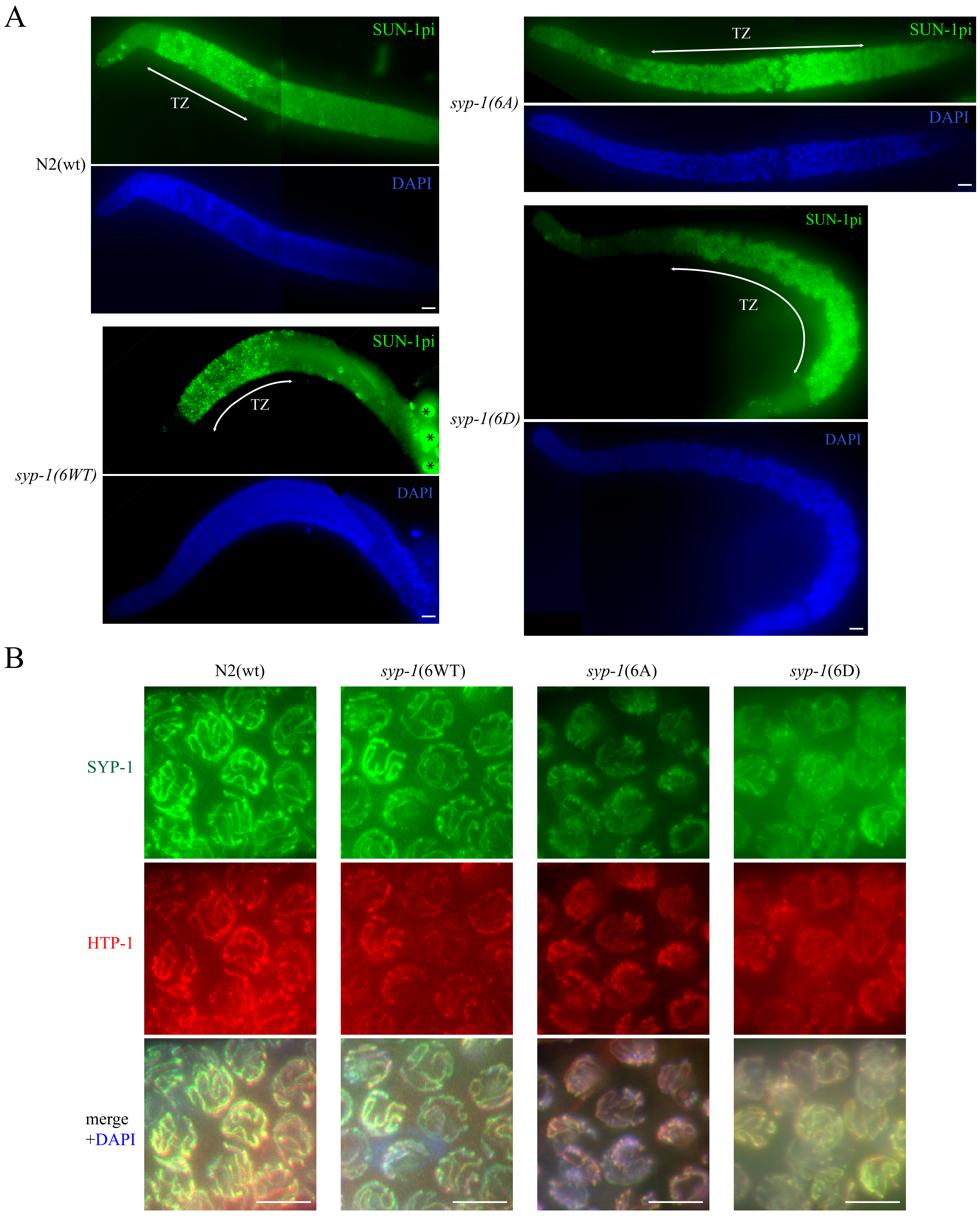

Figure S4. Synaptonemal Complex (SC) assembly in the *syp-1* phosphorylation alleles, Related to Figure 3. (A) Representative images of fixed germlines from the indicated strains immunostained with SUN-1pi antibody, marker of transition zone, and counterstained with DAPI. Scale bar 10µm. Images of the complete germilines were stitched together manually from two pictures that overlap. * Strong signal at diakinesis nuclei **(B)** Representative images of the pachytene region from the indicated strains fixed germlines immunostained with SYP-1 and HTP-1 antibodies (central and lateral elements of the SC, respectively) and counterstained with DAPI. Scale bar 5µm.

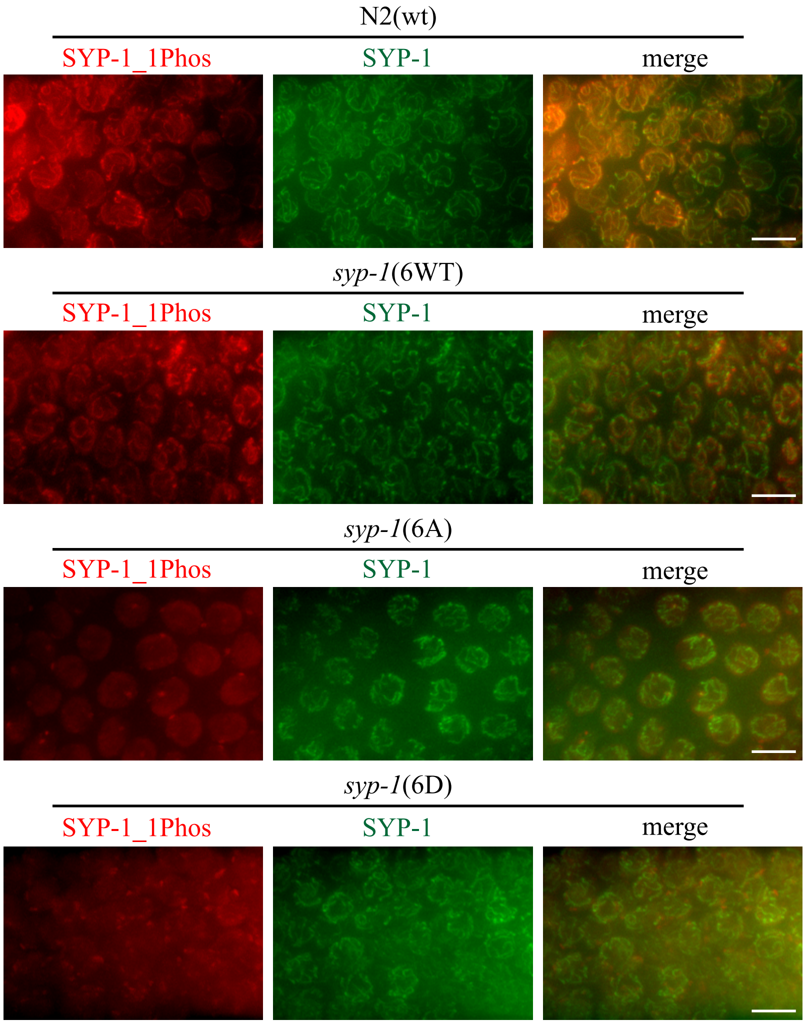

Figure S5. Phospho-antibodies in the *syp-1* phosphorylation alleles, Related to Figure 4. Representative images of the pachytene region from the indicated strains fixed germlines immunostained with SYP-1_1Phos and Synaptonemal Complex protein SYP-1 antibodies and counterstained with DAPI. Scale bar 5µm.

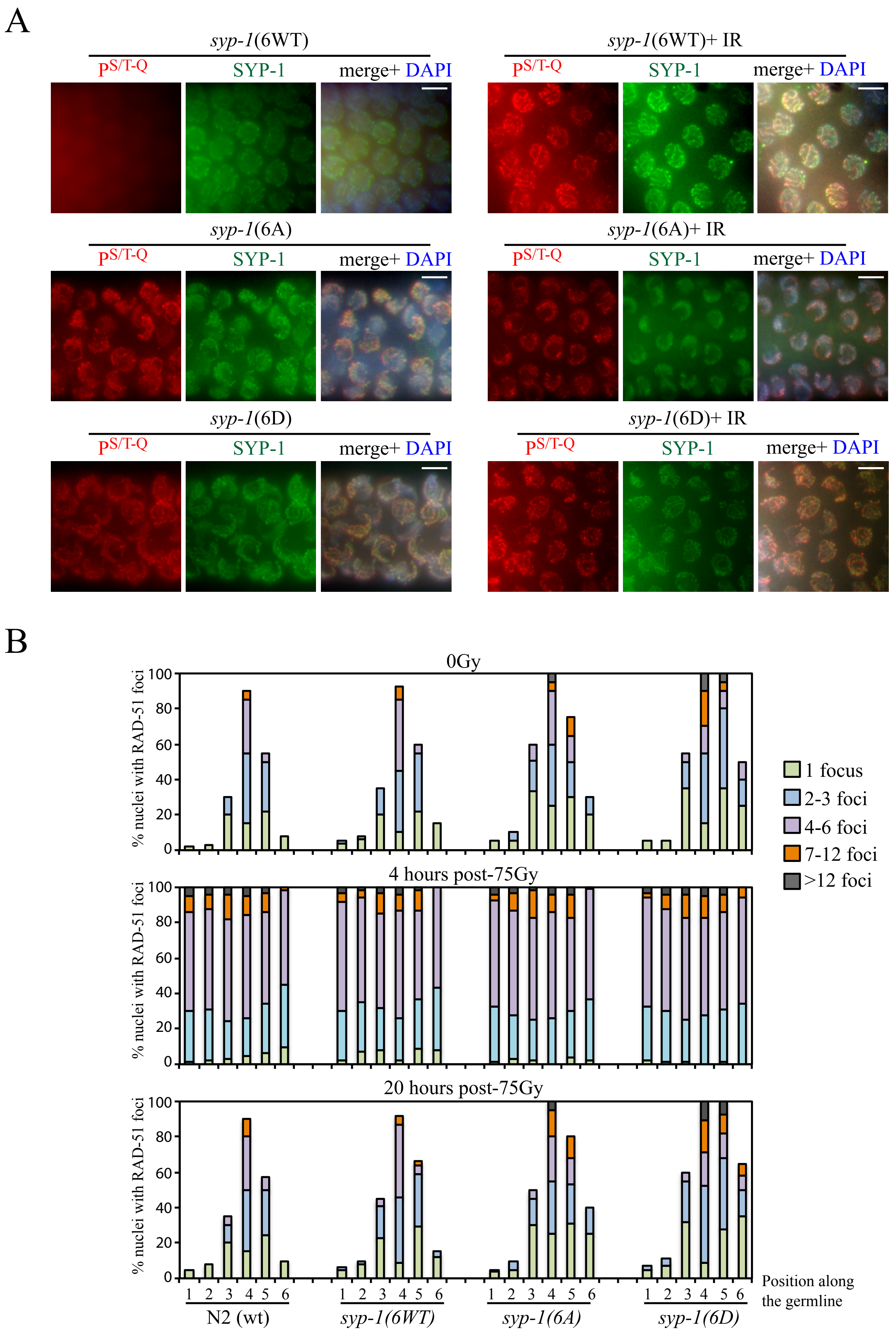

Figure S6. DNA damage response in the *syp-1* phosphorylation alleles, Related to Figure 5. (A) Representative images of the pachytene region from the indicated strains fixed germlines immunostained with anti-$P^{S/T-Q}$ and Synaptonemal Complex protein SYP-1 antibodies counterstained with DAPI without damage (left) or after 1 hour 75Gy (right). Scale bar 5µm. **(B)** Quantitation of RAD-51 focus formation in the indicated strains in normal conditions (top), or 4 (middle) and 20 (bottom) hours post 75Gy. At least fifteen gonads were analysed in each condition and ten nuclei were scored in each zone (mitotic region (1), transition zone (2), early-mid-late pachytene regions (3-4-5) and diplotene-diakinesis regions (6)).

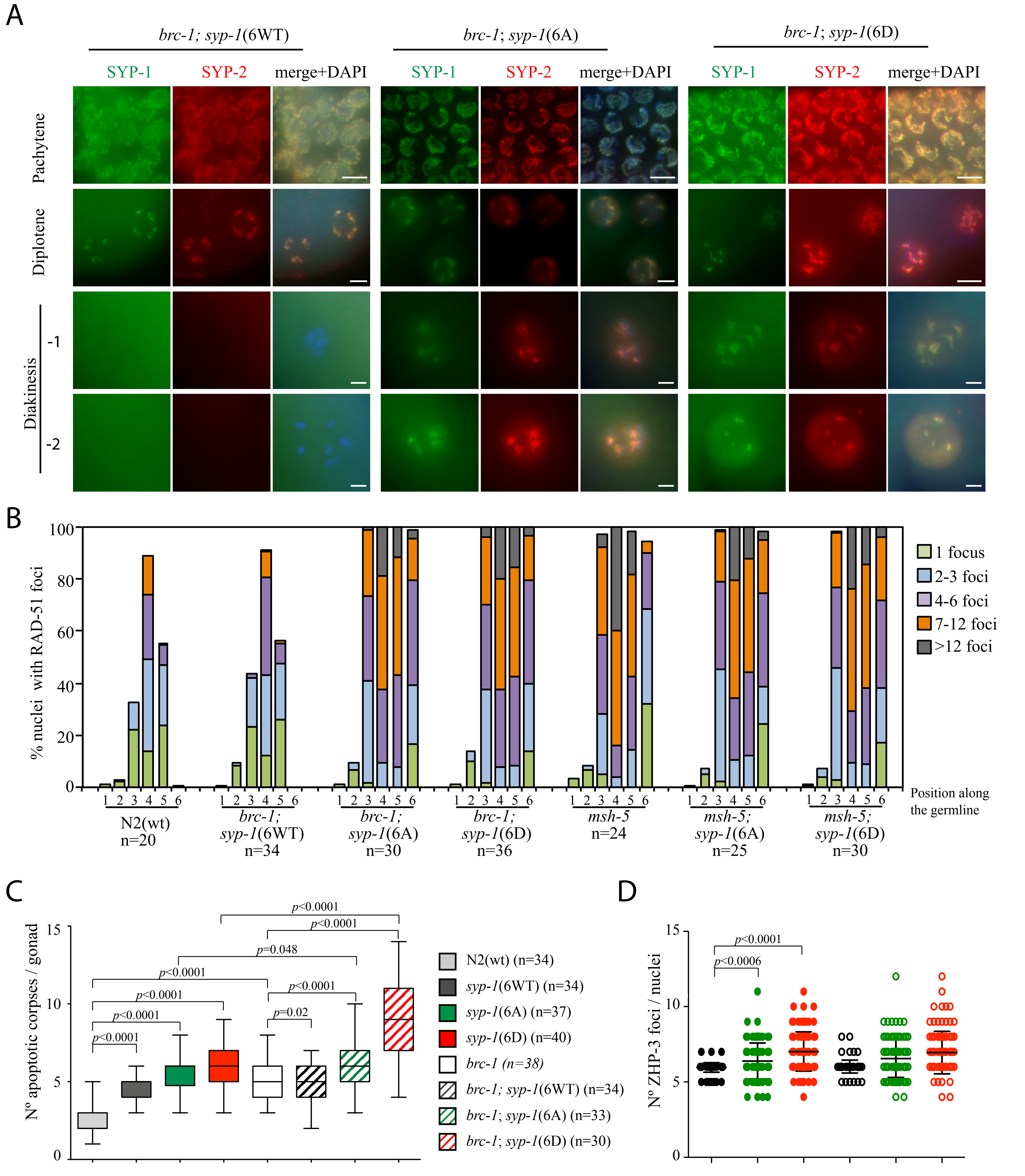

Figure S7. *brc-1; syp-1* phosphorylation alleles, Related to Figure 6. (A) Representative images of the indicated regions from the indicated strains fixed germlines immunostained with Synaptonemal Complex proteins SYP-1 and SYP-2 antibodies counterstained with DAPI. Scale bar 5µm. **(B)** Quantitation of RAD-51 focus formation in the indicated strains. (n) indicates the number of gonads analysed and ten nuclei were scored in each zone (mitotic region (1), transition zone (2), early-mid-late pachytene regions (3-4-5) and diplotene-diakinesis regions (6)). **(C)** Quantation of apoptotic corpses of the indicated strains. (n) indicates the number of animals analysed. Graph shows box and whiskers from minimun to maximun. P value for Paired T-test stadistical analysis is shown. **(D)** Quantation of ZHP-3 foci the indicated strains (120 late pachytene/diplotene nuclei were counted for each strain). Graph shows mean with SD. P value for Paired T-test stadistical analysis is shown.

Table S1. List of primers used in this study, Related to STAR Methods.

Primer	Sequence (5'-3')	Use
SYP1mos1.GWF	GGGGACAAGTTTGTACAAAAAAGCAGGCTGGGAATTTTGC GTATTTTCAAATTT	Cloning
SYP1mos1ter.GWR	GGGGACCACTTTGTACAAGAAAGCTGGGTAAACAGCGATT TTCAAATGTCCTGAT	Cloning
SYP-1site3&4-A-F	GTATTCTCGGCGCCATTGATGGCGGCCGCACCACTTGCCGC AGCTGCCAGACCGTTGAAACGAGCTCGTGCGGCCGATCG	Cloning
SYP-1site3&4-A-R	CGATCGGCCGCACGAGCTCGTTTCAACGGTCTGGCAGCTGC GGCAAGTGGTGCGGCCGCCATCAATGGCGCCGAGAATAC	Cloning
SYP-1site3&4-D-F	TTCTCGGCGCCATTGATGGATGACGATCCACTTGACGCAGC TGACAGACCGTTGAAACGAGATCGTGCGGCCGAT	Cloning
SYP-1site3&4-D-R	ATCGGCCGCACGATCTCGTTTCAACGGTCTGTCAGCTGCGT CAAGTGGATCGTCATCCATCAATGGCGCCGAGAA	Cloning
SYP-1 SQ1	CGGTGTTTGCGGCCTCCGCTC	Sequencing & genotyping
SYP-1 SQ3	GAATTGCAACTTGTCTCTGTC	Sequencing
SYP-1 SQ5	AGCAAATGAGGGAAGATTGCG	Sequencing & genotyping
SYP-1 SQ7	ACTATGATAAGCAAATCAAGG	Sequencing & genotyping
Ti5605E1	ATTCTTGAAGACGACGAGCC	Genotyping
Ti5605E2	TTTTCTCAGTTGTGATACGG	Genotyping
Ti5605E3	CACATCTTTTCTGGCTCTGC	Genotyping
Ti5605E4	TTCAGCCAAGTTACAGGCCG	Genotyping
Ti5605I1	TCAGAGCATTGCTTATCTCG	Genotyping
Ti5605I2	TGTAGTTATACAGAAGACCG	Genotyping
Ti5605I3	TCTGTAAAATATTATCATGG	Genotyping

Ti5605I4	GATCAGCCTCGTTGTCGACC	Genotyping
Cb unc-119 F	GCCGAGCAACAACAATCGATTCCACCCGGC	Genotyping
Cb unc-119 R	GCTTTGTTGTGCATGACGAGTTTGTTATCG	Genotyping
SYP-1 SQ-E1	AGGCAATCTGCAAATTTGCG	Genotyping
SYP-1 SQ-E2	CTGAGAACGGAGTGTGACGC	Genotyping
SYP-1 SQ2	AGTTTTCCCTCTTCGAGCGC	Genotyping
BRC-1 E1	CTCCGTAGCTTGAAGTCTCA	Genotyping
BRC-1 E2	TTTCGGTGGCGCCACATGGA	Genotyping
BRC-1 I1	AATATAGGCACCGGCGGGGA	Genotyping
BRC-1 I2	TGTCGCATCGTCGGCATTAA	Genotyping
BRC-1 E3	CCAGTGGTATGCTTTTTGGC	Genotyping
BRC-1 I3	CTGACTGAAAATCATAGCGG	Genotyping
MSH-5 I1	AGCTTGACCGCCGTCGCCTTACCCCG	Genotyping
MSH-5 I2	TCATCGAACTGTCATGGGACCCGGCC	Genotyping
MSH-5 I3	CGATATTTCAGAAGATCTTG	Genotyping
MSH-5 I4	CCTCTGATGTTCCTTCTTCTG	Genotyping
